# Fe-MOF@Quercetin complex with light-controlled redox modulation for tumor wound repair

**DOI:** 10.1016/j.mtbio.2026.103484

**Published:** 2026-07-21

**Authors:** Yuze Xu, Lan Chang, Zhaowenbin Zhang, Jiang Chang

**Affiliations:** a State Key Laboratory of Advanced Fiber Materials, College of Biological Science and Medical Engineering, Donghua University, Shanghai, 201620, China; b State Key Laboratory of High-Performance Ceramics and Superfine Microstructure, Shanghai Institute of Ceramics, Chinese Academy of Sciences, Shanghai, 200050, China; c Joint Centre of Translational Medicine, The First Affiliated Hospital of Wenzhou Medical University, Wenzhou, 325000, China; d Zhejiang Engineering Research Center for Tissue Repair Materials, Wenzhou Institute, University of Chinese Academy of Sciences, Wenzhou, 325000, China; e Department of Cardiovascular Surgery, Ruijin Hospital, Shanghai Jiaotong University School of Medicine, Shanghai, 200025, China

**Keywords:** Excision of tumor, MIL-101-(Fe)@Quercetin, Oxygen vacancies, Narrow bandgap, Light-controlled redox regulation

## Abstract

After the excision of tumor such as melanoma, the wound site often suffers from tumor recurrence and poor healing. A major clinical dilemma associated with photodynamic therapy is that the tumor excision site requires high reactive oxygen species (ROS) to inhibit tumor recurrence, while low ROS condition is required for promoting wound healing. To solve this problem, our study synthesized a MIL-101-(Fe)@Quercetin (MQ) complex material with oxygen vacancies and narrow bandgap characteristics, aiming to achieve light-controlled redox regulation of ROS generation. The structure of MQ enables it to efficiently separate holes (h^+^) and electron pairs (e^−^) under laser irradiation, thereby generating ROS. At the same time, as a MOF- polyphenol complex, MQ exhibits excellent reducibility and biological activity, which can not only scavenge excess ROS after laser irradiation but also promote angiogenesis. *In vivo* experiments using mouse melanoma wound model demonstrate that MQ Microneedles (MQ@MNs), through light-controlled release of ROS in the tumor area, effectively inhibit tumor growth while scavenging ROS in the wound area to promote skin wound healing. In summary, this study provides new ideas and directions for the application of light-controlled redox regulation of ROS antioxidants in the treatment of melanoma wounds.

## Graphical abstract

**Figure:**
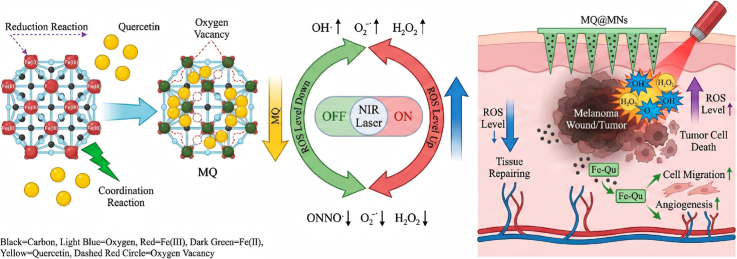

## 1 Introduction

Melanoma is a prevalent skin malignancy, and surgical resection is frequently employed in clinical practice for its treatment. However, the extensive skin wounds formed after surgical resection of melanoma have progressively become another challenging treatment issue in clinical practice. On one hand, melanoma cells often penetrate deep into the dermis, extending beyond most observable lesion areas, making incomplete removal a common issue, particularly for patients with advanced melanoma in stages III-IV [[1], [2], [3]]. These incompletely excised melanoma cells may rapidly proliferate, occupy the growth space of normal skin cells, and cause the wound to further expand and form a melanoma wound [[4], [5], [6]]. On the other hand, although treatments such as chemodynamic therapy (CDT), photodynamic therapy (PDT), and sonodynamic therapy (SDT) may be applied post-surgery to further eliminate residual melanoma and inhibit tumor recurrence, these methods may affect skin wound healing [[7], [8], [9]], and develop to refractory wounds that cannot be completely repaired in both structure and function within the expected time after receiving standardized systemic treatment. Long-term unhealed large-area skin wounds produce a large amount of hydrogen peroxide (H_2_O_2_), leading to a state of strong oxidative stress. Excessive reactive oxygen species (ROS) generated within the wound area can cause chronic inflammation, and thereby inhibit the healing of the skin wound [10,11].

Therefore, it is crucial to develop wound dressings with dual functionalities including inhibition of tumor growth and enhancement of wound healing. Previous studies have revealed that ROS (•OH, ^1^O_2_, and •O_2_^−^) can effectively kill tumors, and ROS-generating materials hold potential for tumor treatment [12,13]. However, the generation of these ROS can conversely inhibit wound healing [14]. Thus, for tumor wound treatment, the challenge in designing functional wound dressings lies in achieving controllable ROS release post-surgery to further eliminate residual tumor cells, while scavenging excess ROS to promote wound healing at later stages.

Metal-organic frameworks (MOFs), such as MIL-101(Fe), have a large number of metal active sites on their surfaces and are commonly used as novel nanomaterials for biomedical applications including photodynamic therapy (PDT) by utilizing their photocatalytic activity to generate ROS [15,16]. However, to enhance catalytic activity, MIL-101(Fe) is often treated with reducing agents such as H_2_ to convert Fe^3+^ to Fe^2+^, thereby creating oxygen vacancy active sites. The synergistic effect of mixed valence states and oxygen vacancies is purported to enhance the catalytic activity of MIL-101(Fe) [[17], [18], [19]]. Furthermore, in order to obtain photosensitivity, MIL-101(Fe) needs to be modified with organic photosensitizers abundant in auxochromes, such as -OH, -NH_2_, and -NR_2_, so that the catalytic activity of the MOF can be activated by infrared laser irradiation, thereby enabling PDT applications [20,21].

Polyphenolic compounds, such as quercetin (Qu), are typical reducing agents [14,22]. Interestingly, many polyphenolic compounds also exhibit chelating activity with a range of metal ions, including Fe^3+^ and Fe^2+^ [[23], [24], [25]]. These metal-polyphenol chelates possess strong reducing properties and exhibit the ability to scavenge ROS [26,27]. Furthermore, some chelates exhibit unique biological activities, and may promote vascular regeneration and tissue repair [[28], [29], [30]]. More intriguingly, polyphenols like Qu, which are rich in auxochrome groups such as -OH, may exhibit good light absorption properties, particularly when they form chelates with metal ions, and function as photosensitizers [31,32]. Recently, the paradigm of wound management and post-surgical therapy has increasingly shifted toward smart biomaterials. These advanced platforms can dynamically sense specific microenvironmental cues to shape pro-regenerative and anti-inflammatory tissue beds, effectively addressing the clinical conflict between localized disease ablation and subsequent tissue repair [33,34]. Concurrently, novel metal-dependent programmed cell death pathways have introduced transformative strategies for localized antibacterial therapy and pathogen eradication [35]. Inspired by these dynamic microenvironment-modulating strategies and considering the aforementioned multi-functional characteristics of polyphenols, we proposed a hypothesis that polyphenols may on one side serve as reducing agents to prepare metal-organic frameworks (MOFs) with high catalytic activity, and on the other side chelate with Fe ions in MIL-101(Fe), endowing them with high photosensitivity for light-controlled ROS generation to eliminate tumor cells, as well as ROS scavenging and bioactivity for enhancing wound healing.

**Scheme 1:**
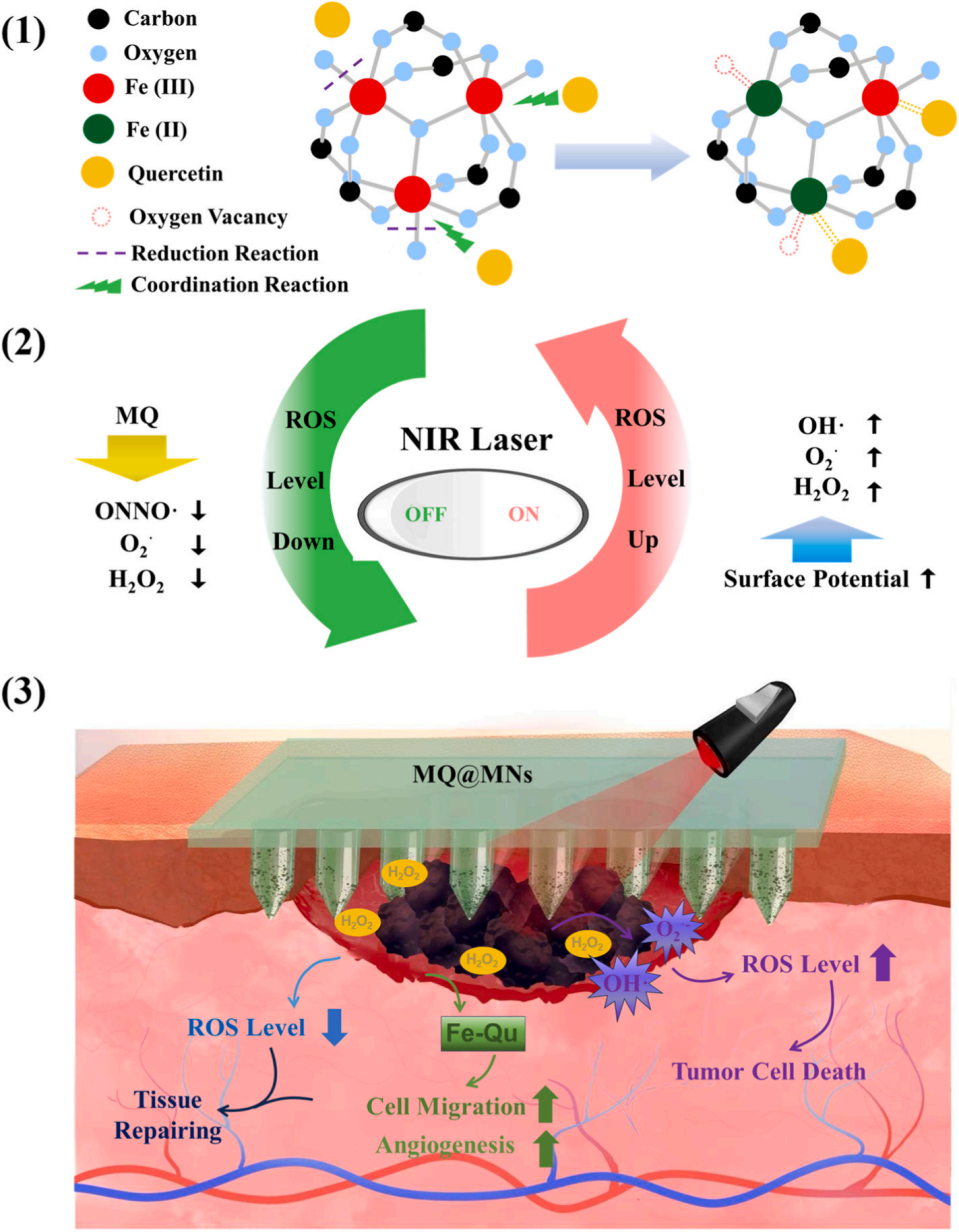
**Possible Reactions of MIL-101(Fe) and Qu, the Function of Reaction Products (MQ), and treatment of tumor wound with Qu composite microneedles. (1) Microscopic Schematic Diagram of the Synthesis of MQ:** Under the influence of Qu, a coordination reaction occurs between Qu and the Fe^3+^ active sites in the metal-organic framework (MOF), leading to the formation of an Fe-Qu complex. During this process, Fe^3+^ is reduced to Fe^2+^, resulting in the generation of oxygen vacancies (Ov). **(2) Laser Controlled Redox Modulation of MQ for ROS Generation/Scavenging:** When laser irradiation is applied, the surface potential of MQ increases, leading to the generation of ROS (•OH, •O_2_^−^, and H_2_O_2_). Conversely, laser irradiation switched off, MQ reveals reducing property and scavenges ROS (H_2_O_2_, •ONOO^−^, •O_2_^−^). **(3) Treating Melanoma Wounds using MQ Composite Microneedles (MQ@MNs) with Light Controlled Redox Modulation:** MQ@MNs release MQ particles into the melanoma wound area. When the laser is turned on, MQ@MNs in the tumor area generates ROS and induces tumor cell apoptosis. When the laser is turned off, MQ@MNs scavenges ROS in the wound area and mitigates the effects of oxidative stress. Simultaneously, the degradation of MQ releases bioactive Fe-Qu chelates that facilitate cell migration and angiogenesis.

To test our hypothesis, we designed a reaction using Qu and MIL-101(Fe) with two possible reaction processes to synthesize a novel Fe-MOF@Quercetin complex. Firstly, Qu reduces Fe^3+^ in MIL-101(Fe) to form oxygen vacancy active sites. Secondly, Qu coordinates with Fe^3+^/Fe^2+^ in MIL-101(Fe). The redox and photocatalytic properties of the products (MQ), particularly their light-controlled ROS generating and scavenging properties, were evaluated. Furthermore, MQ/sodium hyaluronate composite microneedles were prepared and the effect in eliminating melanoma and enhancing the healing of skin tumor resection wounds was investigated using a mouse melanoma wound model (Scheme 1).

## 2 Results and discussion

### 2.1 Preparation and Characterization of MIL-101(Fe)/Qu complex (MQ)

**Fig. 1:**
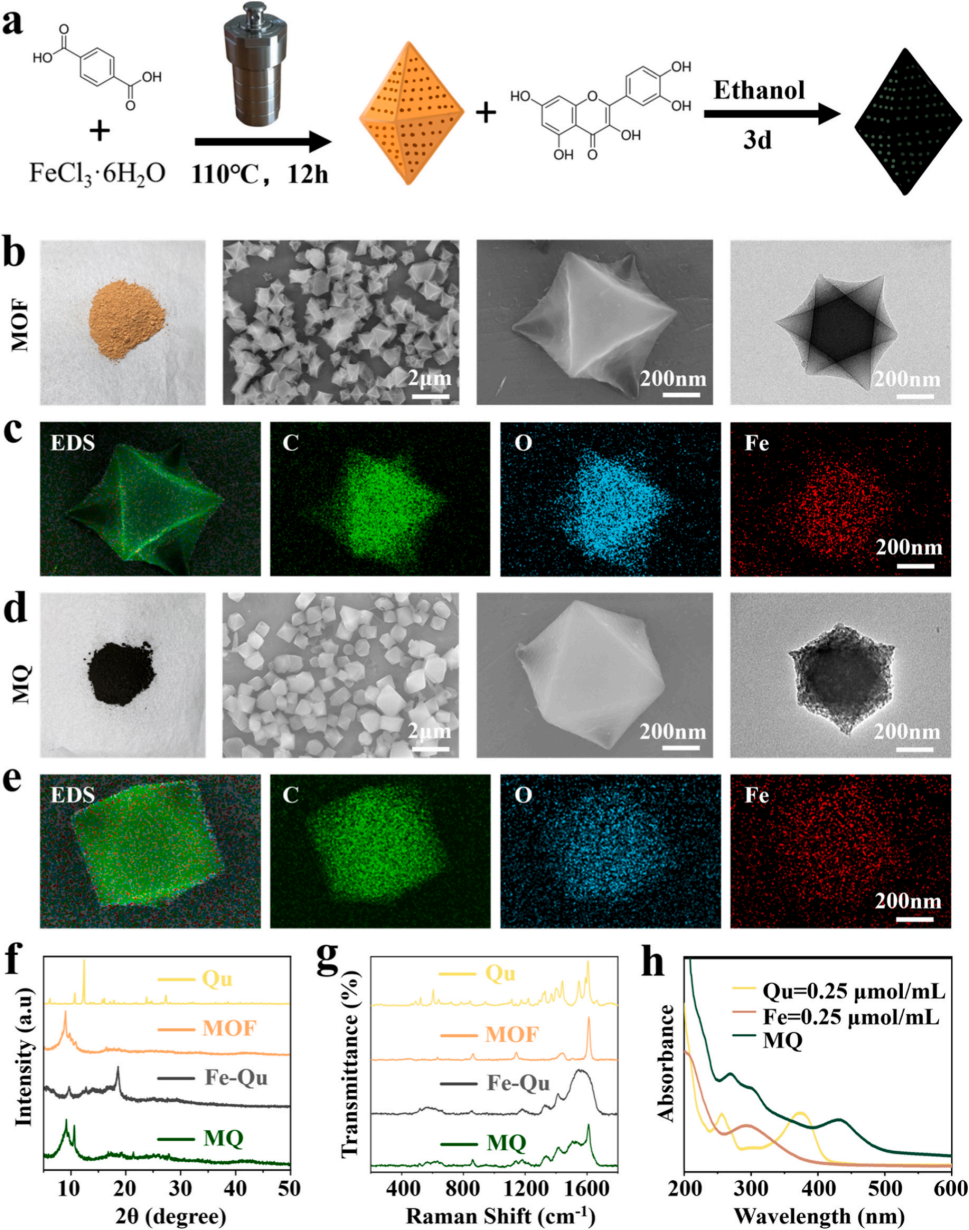
**Preparation and Characterization of MIL-101 (Fe)/Qu (MQ).** (a) Schematic illustration of the synthesis route for MQ. (b) Macroscopic optical image, SEM image, and TEM image of MOF powder. (c) Elemental distribution mapping of MOF powder. (d) Macroscopic optical image, SEM image, and TEM image of MQ powder. (e) Elemental distribution mapping of MQ powder. (f) XRD patterns of Qu, MOF, Fe-Qu, and MQ. (g) Raman spectra of Qu, MOF, Fe-Qu, and MQ. (h) UV-Vis spectra of MQ particle extracts and Qu-Fe^3+^ solutions.

We first successfully prepared typical iron-based metal-organic framework (MOF) MIL-101(Fe) particles based on reported method with appropriate modification [36]. Subsequently, these MOF particles were reacted with Qu in ethanol solution, and benefiting from the abundant active Fe^3+^ sites in the MOF, which can react with the phenolic hydroxyl groups in Qu [37], MOF@Qu (MQ) complex was successfully synthesized (Fig. 1a). Macroscopically, the prepared MOF appears orange, while scanning electron microscope (SEM) imaging reveals that microscopically, the MOF has an octahedral structure with smooth surface (Fig. 1b). The EDS results show uniform distributed C, O and Fe elements in obtained MOF particles (Fig. 1c). After reacting with Qu, the product MQ appears noticeably black macroscopically, indicating that the reaction of Qu with MOF occurred. The transmission electron microscope (TEM) image further shows that the internal structure of MQ becomes rougher compared to the MOF, which further confirms the reaction of MOF with Qu (Fig. 1d). The EDS results of MQ indicate that the reaction with Qu did not affect the overall distribution of the elemental composition of the MOF (Fig. 1e). Dynamic light scattering (DLS) and Zeta potential measurements further confirmed the successful coordination reaction. Compared to pristine MOF, the hydrodynamic diameter of MQ increased from 450 to 530 nm, attributed to the steric hindrance and hydration layer expansion induced by grafted polyphenols. Concurrently, its zeta potential fundamentally inverted from positive (+25 to +30 mV) to negative (−12 to −18 mV). This polarity reversal mechanistically verified the coordination between Qu's phenolic hydroxyls and exposed Fe^3+^ nodes, which effectively shielded the positive charge and enhanced the colloidal stability via electrostatic repulsion (Figs. S1 and S2).

To investigate the specific structure of MQ, we conducted X-ray powder diffraction (XRD) analysis (Fig. 1f). As compared with the XRD pattern of Qu, MOF and free Fe-Qu chelates, it is interesting to see that the MQ composite still retained the characteristic peaks typical of MOF. However, the two main characteristic peaks of Qu at 2θ angles of 12.4° and 10.2° disappeared, and a new peak appeared at 2θ = 10.6°. This discovery indicates that a reaction occurred between MOF and Qu, leading to the disruption of the originally regular lattice structure and subsequently causing the characteristic peaks of Qu to broaden and merge with each other [38,39]. Furthermore, when we compare the XRD pattern of MQ with that of the Fe-Qu chelates, the pattern for MQ does not contain characteristic peaks for free Fe-Qu chelates. This result further confirms that a coordination reaction occurred between the MOF and Qu, and MQ does not contain adsorbed Fe-Qu chelates. In addition to XRD analysis, Raman spectroscopy analysis (Fig. 1g) showed that the spectrum of MQ contained peaks of both MOF and Fe-Qu [40], indicating that the molecular structures of both MOF and Fe-Qu are present in MQ. However, the XRD pattern of MQ did not show any characteristic peaks of the Fe-Qu chelate, which suggests that Qu is coordinated with Fe^3+^ on the surface of the MOF and forms a complex with structural characteristics peaks of Fe-Qu chelate bonds in Raman spectrum, but no free Fe-Qu chelates exist.

To further verify the interaction between Qu and the MOF, we used UV-Vis spectroscopy to analyze the degradation products of MQ after immersion in deionized water for 24 h and compared the ultraviolet absorption spectrum between MQ, Qu and Fe^3+^ (Fig. 1h and Fig. S3). The results showed that MQ degradation products exhibited a characteristic peak similar as Fe-Qu chelates at 440 [41] nm and no characteristic peaks of Qu and Fe^3+^ at 374 nm and 290 nm, suggesting that the MQ degradation products contain free Fe-Qu chelates, although MQ does not show the characteristic XRD spectrum of free Fe-Qu chelates.

For investigation of the degradation and release behavior of MQ in physiological environment, we employed Inductively Coupled Plasma Spectroscopy (ICP) and UV-Vis spectroscopy to analyze the degradation products of MQ in simulated tumor weak acid microenvironment and wound neutral microenvironment. As depicted in Fig. S4, the degradation performance of both MOF and MQ was significantly affected by different physiological conditions, and MQ demonstrated a higher release rate of Fe ions as compared to MOF. These Fe ions encompassed Fe^3+^ and Fe-Qu chelates generated from MQ degradation. This result indicates that coordination with Qu enhanced the degradation performance of MOF, and the acid environment further promoted the release of Fe ions from both MOF and MQ. Fig. S5 revealed a similar trend, that the release amount of Fe-Qu increased with time.

### 2.2 MQ promote photocatalytic performance and long-lasting reducibility

**Fig. 2:**
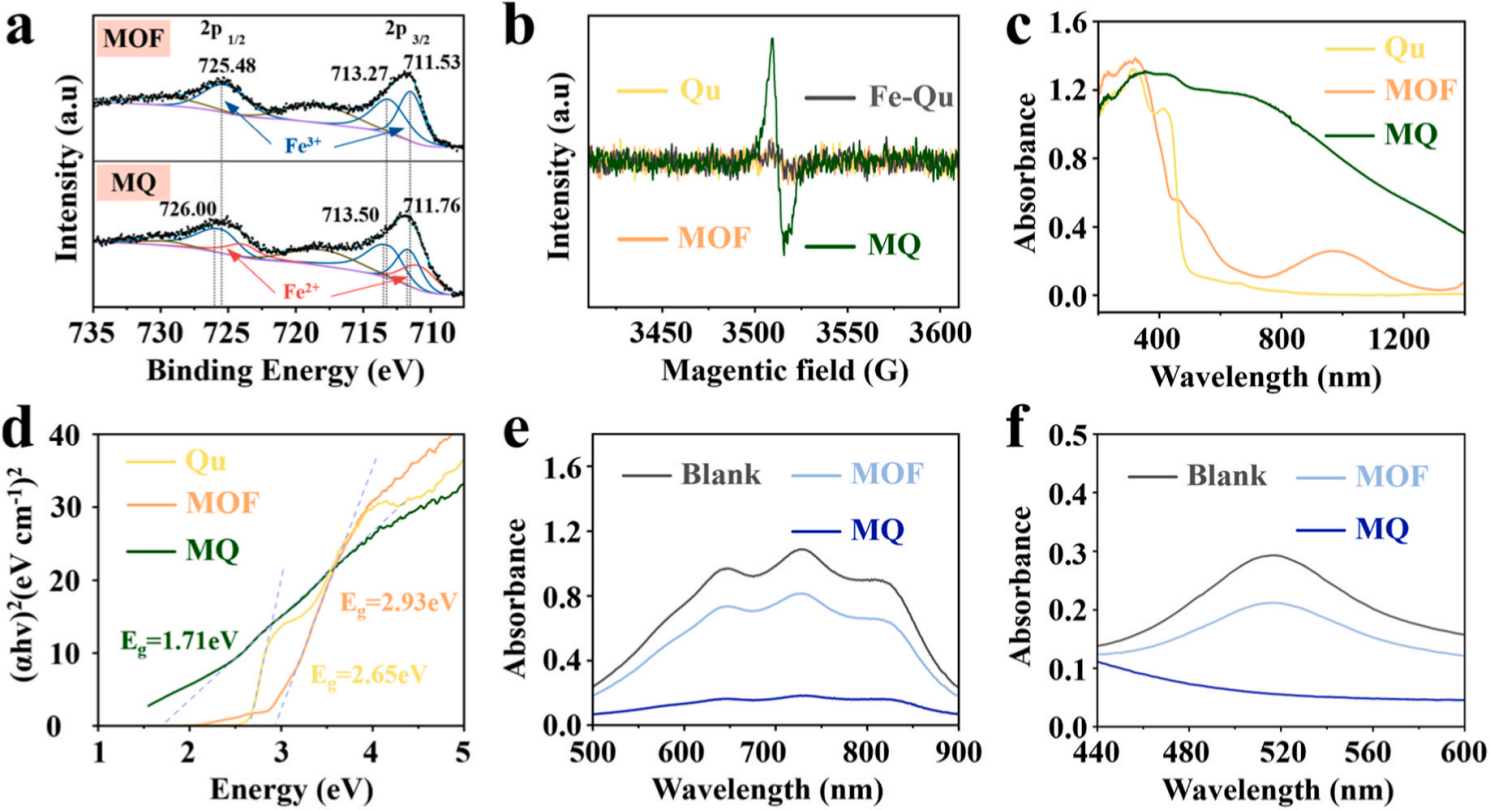
**Enhanced Photocatalytic Performance and Long-term Reducibility of MQ Derived from MOF coordinated Qu.** (a) XPS spectra (Fe 2p peaks) of MOF and MQ to evaluate the Fe^3+^/Fe^2+^ mixed valence. (b) EPR spectra of Qu, MOF, Fe-Qu, and MQ to assess oxygen vacancies. (c) UV-Vis-DRS diffuse reflectance spectra of Qu, MOF, and MQ to evaluate light absorption capacity. (d) Optical bandgap diagrams of MOF and MQ to assess bandgap values. (e) UV-Vis spectra of ABTS solutions containing MOF and MQ to evaluate reducibility (Blank represents the ABTS solution without MOF or MQ). (f) UV-Vis spectra of DPPH solutions containing MOF and MQ to evaluate reducibility (Blank represents the DPPH solution without MOF or MQ).

We utilized X-ray Photoelectron Spectroscopy (XPS) to determine the electronic state and chemical composition of MQ. The XPS spectra of MOF and MQ revealed prominent measurement signals for C, O, and Fe elements, indicating that the element types remained unchanged before and after the reaction (Fig. S6). Subsequently, peak splitting was performed for the C, O, and Fe elements in MOF and MQ. As shown in the C, O element spectrum in Fig. S7a and S7b, the additional C-O and CO-H bonds (red lines) are observed in MQ as compared to MOF, which are possibly from the phenolic hydroxyl groups of Qu [42]. Interestingly, compared to MOF, the Fe-O binding energy in MQ decreases from 531.92 eV to 531.80 eV in Fig. S7b, indicating the probable existence of interaction between Qu and Fe in MOF. Furthermore, the Fe 2p states analysis reveals that, as compared to MOF, the binding energies of Fe 2p_3/2_ and Fe 2p_1/2_ in MQ are both elevated, and in addition to the original Fe^3+^ signal peak (blue lines), the Fe^2+^ signal peak (red lines) appeared (Fig. 2a). According to band theory [43,44], above change can be attributed to the coordination between Fe^3+^ in MOF and phenolic hydroxyl groups in Qu. Since the electronegativity of O is greater than that of Fe, the electron cloud density around Fe decreases after coordination, leading to an increase in the binding energy of Fe 2p [45,46]. Simultaneously, the appearance of an Fe^2+^ peak in the MQ XPS spectrum indicates that some Fe^3+^ in MOF are reduced to Fe^2+^ by Qu. This process results in the breakage of Fe^3+^-O bonds, allowing oxygen atoms to escape and form oxygen vacancies [47]. Furthermore, the transfer of electrons creates an internal electric field between MOF and Qu with the presence of both Fe^3+^ and Fe^2+^ [48]. This mixed valence state and internal electric field effectively promote the movement and separation of photogenerated carriers.

To further verify the formation of oxygen vacancies (O_V_), MQ, MOF, Qu and Fe-Qu were analyzed using Electron Paramagnetic Resonance (EPR) spectroscopy. As shown in Fig. 2b, MOF, Qu, and the Fe-Qu chelate do not exhibit signal peaks for O_V_, while MQ shows a strong signal peak for O_V_. This result further confirms that Fe^3+^ in MOF is reduced to Fe^2+^ by Qu, leading to the breakage of Fe-O bonds. The formed O_V_ is beneficial to improve the light absorption range (red shift) of MQ and enhance its light absorption performance [49,50].

Optical properties play a crucial role in the photocatalytic activity of materials. Metal-polyphenol chelates are believed to narrow the bandgap of materials and enhance photochemical activity [51]. UV-Vis diffuse reflectance spectroscopy (UV-Vis-DRS) reveals that MQ exhibits significantly higher light absorption capability in the infrared region than Qu and MOF (Fig. 2c). This result suggests that, after the coordination reaction between MOF and Qu, the organic groups of Qu act as auxochromes [[52], [53], [54]], causing a red shift in the absorption peak of MOF and endowing MQ with excellent light absorption capability, which makes it suitable for infrared light based PDT therapy. Additionally, the bandgap values calculated using the Tauc Plot function method show that the bandgap values of MOF and Qu are 2.93 eV and 2.65 eV, respectively, while the bandgap value of MQ is 1.71 eV (Fig. 2d) [55]. These results indicate that MQ has better light absorption capability, narrower bandgap, and higher light energy conversion efficiency. Therefore, under 660 nm Laser excitation, MQ can generate holes (h^+^) and electrons (e^−^) on its surface, and the mixed valence state and oxygen vacancies promote the separation of electrons and holes [39], thereby endowing MQ with excellent photocatalytic performance [56].

Metal-polyphenol chelates are considered a type of strong reductive material [57]. Given that MQ is a complex formed by the coordination between MOF and Qu, we speculate that MQ possesses reducibility. By detecting the content of ABTS and DPPH free radicals in solution, we evaluated the reducibility of the material. The light absorption spectra shown in Fig. 2e and f indicate that MOF itself does not possess high reducibility [58] and can only remove limited amount of ABTS and DPPH through physical adsorption. In contrast, MQ exhibits much stronger reducibility as compared to MOF, with significantly reduced content of ABTS and DPPH in the solution and notably weakened spectral absorption. Based on the standard curve of Qu (Fig. S8) and the change in Qu concentration before and after the reaction between MOF and Qu, we calculated the amount of Qu coordinated with MOF, and found 8.265 mg Qu in 47.248 mg MQ, and the ratio of the formed complex MOF to Qu is 2:1 (Table S1). Subsequently, we compared the reducibility of MQ and Qu, and the result shows that, within 0.5 h after adding Qu, the ABTS and DPPH in the solution were quickly removed. However, the removal of ABTS and DPPH stopped after 0.5 h because Qu has been fully consumed. In contrast, MQ can continuously remove free radicals within 6 h because the reducibility of this complex is stronger than that of Qu (Figure S9a and Figure S9b). In summary, MQ formed after coordination between MOF and Qu exhibits excellent long-lasting reducibility.

### 2.3 Light controlled generation and scavenging of ROS by MQ with/without laser irradiation

Given photocatalytic potential of MQ, it undergoes electron(e^−^)-hole(h^+^) separation under laser irradiation. These separated h^+^ and e^−^ can react with H_2_O and O_2_, respectively, generating hydroxyl radicals (•OH) and superoxide anions (•O_2_^−^) with strong oxidizing properties [59]. •O_2_^−^ can combine with H_2_O and convert into H_2_O_2_ [60], while MOF, acting as a peroxidase-like enzyme (POD), catalyzes H_2_O_2_ to generate •OH [61].

In this study, we aimed to achieve light-controlled redox regulation of ROS release, so the light-controlled performance of generation and scavenging of ROS is evaluated. It is well known that H_2_O_2_, •ONOO^−^, and •O_2_^−^ are three typical radicals that cause ROS damage to tissues and organs [62]. To delineate the compositional dependency of the light-controlled redox switch, the H_2_O_2_ clearance efficiency of the complex at varied MOF:Qu ratios was evaluated (Fig. S10). Under dark conditions, the clearance efficiency exhibits a Qu-concentration-dependent escalation, reaching near-complete H_2_O_2_ scavenging (97.54 ± 2.30%) at a 2:1 ratio and plateauing thereafter. Conversely, under laser irradiation, the photogenerated electron-hole pairs trigger an oxidative burst that competitively neutralizes the inherent reducing capacity of the complex. Crucially, the 2:1 ratio demonstrates the maximum functional contrast. It exhibits robust antioxidant activity in the dark while severely suppressing H_2_O_2_ clearance (14.08 ± 3.58%) under irradiation. Mechanistically, excess Qu (ratios <1:1) acts as a radical sink that excessively quenches the photogenerated reactive species, thereby failing to achieve the desired laser-triggered oxidative state. Conversely, insufficient Qu (ratios >4:1) inadequately scavenges H_2_O_2_ even in the dark. These findings strictly validate that the 2:1 stoichiometric coordination uniquely balances the photodynamic oxidation and polyphenol-mediated reduction, serving as the optimal formulation for reversible redox modulation.

**Fig. 3:**
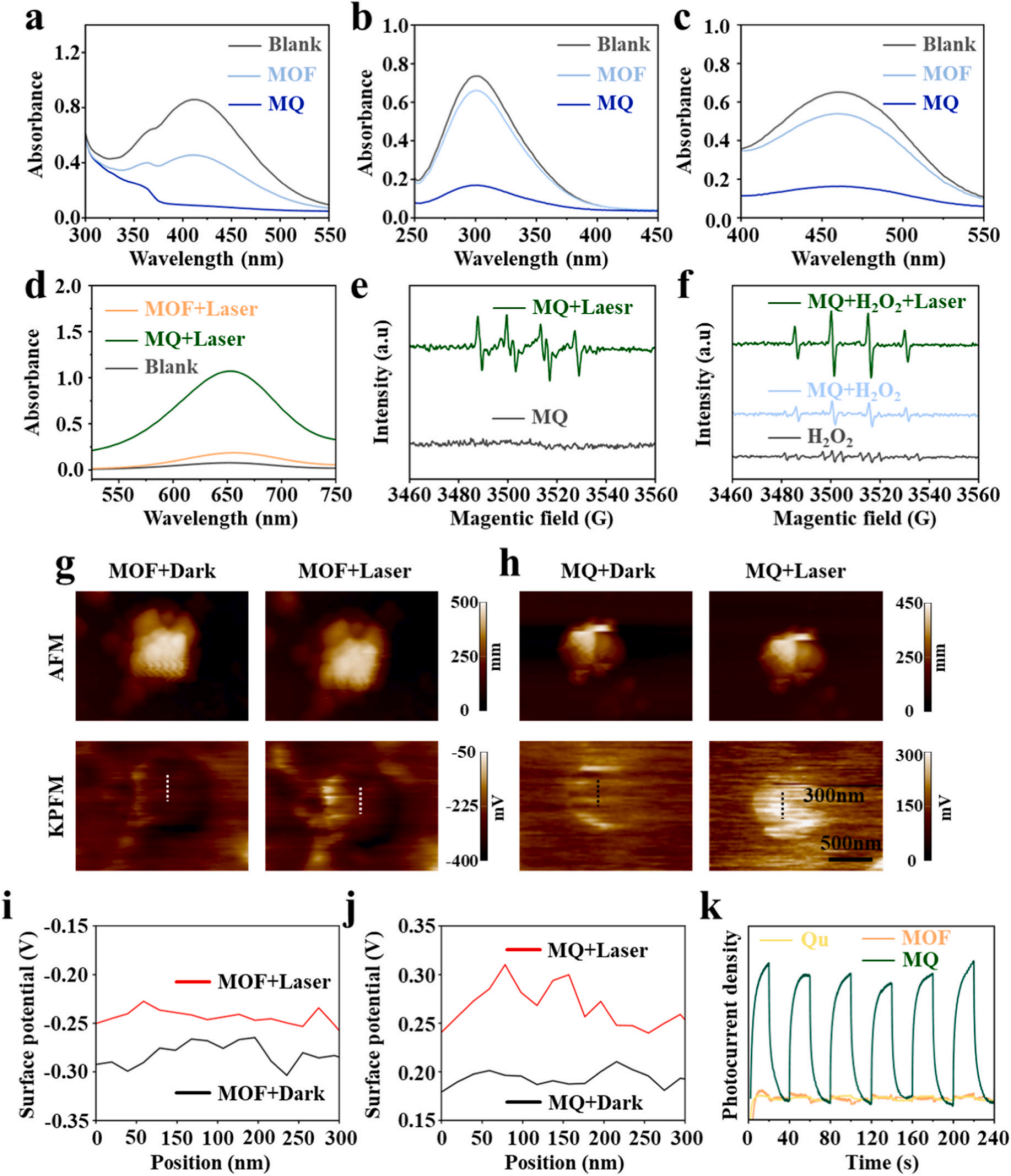
**Photo-modulated bidirectional reactive oxygen species (ROS) regulation and the underlying interfacial charge transfer dynamics of the MQ composite.** (a) Scavenging effects of MOF and MQ on H_2_O_2_. (b) Scavenging effects of MOF and MQ on •OONO. (c)Scavenging effects of MOF and MQ on •O_2_^−^ (Blank represents the ROS solution without MOF or MQ). (d) UV-vis absorption spectrum of •OH generated by the reaction of MOF and MQ with TMB under laser irradiation (sodium acetate buffer, pH = 6.0, 10 min). (e) ESR spectrum of •O_2_^−^ generated by MQ in DMSO under laser irradiation. (f) ESR spectrum of •OH generated by MQ in H_2_O_2_ solution (pH = 6.0) (laser: 660 nm, 0.5W/cm^−2^). (g) Atomic Force Microscopy (AFM) and Kelvin Probe Force Microscopy (KPFM) images of MOF before and after laser irradiation. (h) Atomic Force Microscopy (AFM) and Kelvin Probe Force Microscopy (KPFM) images of MQ before and after laser irradiation. (i) Surface potential changes of MOF before and after laser irradiation. (j) Surface potential changes of MQ before and after laser irradiation. (k) Photocurrent responses of Qu, MOF, and MQ under 660 nm, 0.5 W/cm^−2^ Laser irradiation for assessing the light-controlled ROS generating and scavenging properties.

Furthermore, we used a H_2_O_2_ content assay kit to detect the H_2_O_2_ scavenging ability of MQ under the condition of laser switch-off. It is known that, due to catalase-like (CAT) activity, MOF can catalyze and scavenge H_2_O_2._ As shown in Fig. 3a, MQ shows stronger H_2_O_2_ scavenging effects as compared to MOF, attributed to the mixed valence states of Fe^3+^/Fe^2+^ in MQ [63], which accelerate the catalysis of H_2_O_2_. Subsequently, we measured scavenging ability of MQ on ROS (•ONOO^−^ and •O_2_^−^). As shown in Fig. 3b and c, MQ revealed strong ROS scavenging ability with significant decreases in absorbance values for both two type of ROS, indicating substantial reduction of •ONOO^−^ and •O_2_^−^ in the solution, while Blank and MOF groups exhibit almost no ability to scavenge ROS. This phenomenon can be attributed to the coordination of Qu with Fe in MOF, leading to the formation of a reducing MOF-Qu complex [64].

We then evaluated the generation and scavenging performance of ROS under laser irradiation. We employed the TMB (3,3′,5,5′-Tetramethylbenzidine) assay to assess MQ's ability to generate ROS (•OH and •O_2_^−^) under laser irradiation (660 nm, 0.5 W/cm^−2^). Fig. 3d shows the UV-vis absorption of TMB over 10 min across different groups. The results reveal that MQ exhibits a significant absorption peak at 652 nm under laser irradiation, while Blank and MOF groups do not have, suggesting that MQ undergoes electron-hole separation under laser irradiation, thereby generating ROS (•OH and •O_2_^−^). The generated •O_2_^−^ further reacts with H_2_O to produce H_2_O_2_, which subsequently reacts again with TMB to generate •OH under catalysis of MQ.

To delve into the nature of the types of radicals generated, we used 5,5-dimethyl-1-pyrroline-N-oxide (DMPO) as a radical scavenger and employed electron spin resonance (ESR) technology to detect the types of ROS generated. As shown in Fig. 3e, in a DMSO environment without H_2_O, MQ produces a characteristic sextuplet peak signal under laser irradiation, indicating that MQ can catalyze O_2_ to generate •O_2_^−^, which is captured by DMPO. The results in Fig. 3f reveal that MQ produces a typical quadruplet peak signal belonging to •OH in the presence of H_2_O_2_. This is because MQ can function as a POD enzyme in a weakly acidic environment, catalyzing H_2_O_2_ to generate •OH. Notably, the quadruplet peak signal is enhanced under laser irradiation, indicating that photocatalytic ability of MQ enhances the conversion of H_2_O to •OH within the system. The signal peak observed in the H_2_O_2_ solution without any added material is due to the influence of carboxyl groups in sodium acetate buffer. These results demonstrate MQ robust ability to generate •OH and •O_2_^−^, which stems from the synergistic effects of MOF-polyphenol complexes and oxygen vacancies in MQ, leading to a narrow bandgap and making it easier for electron-hole separation to occur under laser irradiation. On one hand, the holes possess oxidizing properties, enabling the conversion of H_2_O to •OH. On the other hand, the electrons possess reducing properties, enabling the conversion of O_2_ to •O_2_^−^. Furthermore, the mixed valence states of Fe^3+^/Fe^2+^ in MQ promote the mobility and separation efficiency of photogenerated carriers, thereby enhancing photodynamic efficiency.

To rigorously delineate the photocatalytic ROS generation mechanism of the MQ complex, the potential production of singlet oxygen (_1_O^2^) was further investigated. The fluorescence intensity (SOSG) of the _1_O^2^ exhibited that the MQ + Laser group maintained a baseline fluorescence level statistically identical to both the Blank and dark MQ groups, validating the absence of _1_O^2^ generation (Fig. S11). Consistently, corroborative electron spin resonance (ESR) analysis (Fig. S12) revealed a completely flat baseline spectrum without any characteristic _1_O^2^ trapping signals under continuous laser excitation. These analytical results unambiguously exclude the Type-II energy transfer pathway. Taken together with the aforementioned DMPO-ESR data, it explicitly confirms that the laser-triggered ROS burst of the MQ complex is exclusively governed by a Type-I electron-hole separation mechanism.

Furthermore, we used Atomic Force Microscopy (AFM) and Kelvin Probe Force Microscopy (KPFM) to measure the surface potential of MQ under laser irradiation and compared with dark condition. Fig. 3g and h show the AFM and KPFM images of MOF and MQ particles, respectively. The AFM results indicate that the morphology of MOF and MQ, especially the thickness, was not changed, while KPFM results indicate that the surface potential of MQ increases significantly and the surface potential of MOF does not significantly change under Laser irradiation. This phenomenon can be attributed to the narrow bandgap and oxygen vacancy characteristics of MQ, which make it easier for electron-hole separation after Laser irradiation. In the mixed valence state of Fe^3+^/Fe^2+^, electrons are more prone to migrating to the surface of MQ particles and generating holes [65]. The accumulation of these photogenerated holes subsequently leads to an increase in surface potential [66], indicating that MQ is in a state of high oxidative activity. The surface potential distributions of MOF and MQ under dark condition and Laser irradiation are shown in Fig. 3i and j, respectively. Given that the efficiency of ROS generating is closely related to the efficiency of electron-hole separation [67], when the efficiency of ROS generation is higher than the ROS scavenging efficiency, the separation efficiency of electron-hole pairs will be higher than their recombination efficiency. Compared with MOF, MQ generates a significant potential difference under Laser irradiation, indicating that the efficiency of ROS generating by MQ is higher than its scavenging efficiency under this condition and it was in an oxidized state, suggesting that the ROS generated by MQ under laser irradiation would not be cleared by its inherent scavenging properties. To quantitatively verify the net oxidative state under irradiation, the absorbance of the TMB oxidation product at 652 nm was evaluated (Fig. S13). The absorbance value of the MQ + Laser group exhibits a significant elevation compared to the negligible baseline of the dark MQ group. This result validates that the light-triggered ROS generation strictly surpass the scavenging capacity, thereby establishing a net oxidative microenvironment essential for tumor ablation.

Based on the characteristics that MQ exhibits an oxidative state under Laser irradiation and a reducing state under normal conditions, it is observed that Laser acts as a switch to regulate the release of ROS. To detect the electron-hole separation efficiency of MQ, we conducted a photocurrent response experiment. As shown in Fig. 3k, when the Laser switch is turned on, a current signal appears instantly, indicating that MQ is in a state of generating ROS, and when the Laser switch is turned off, the current signal disappears, indicating that MQ is in a state of scavenging ROS. After experiencing six cycles of on-off processes, there is no significant change in current intensity, indicating the stability of the photosensitive performance in generating and scavenging of ROS through light-control.

### 2.4 The light-controlled generation/scavenging of ROS by MQ *in vitro*

**Fig. 4:**
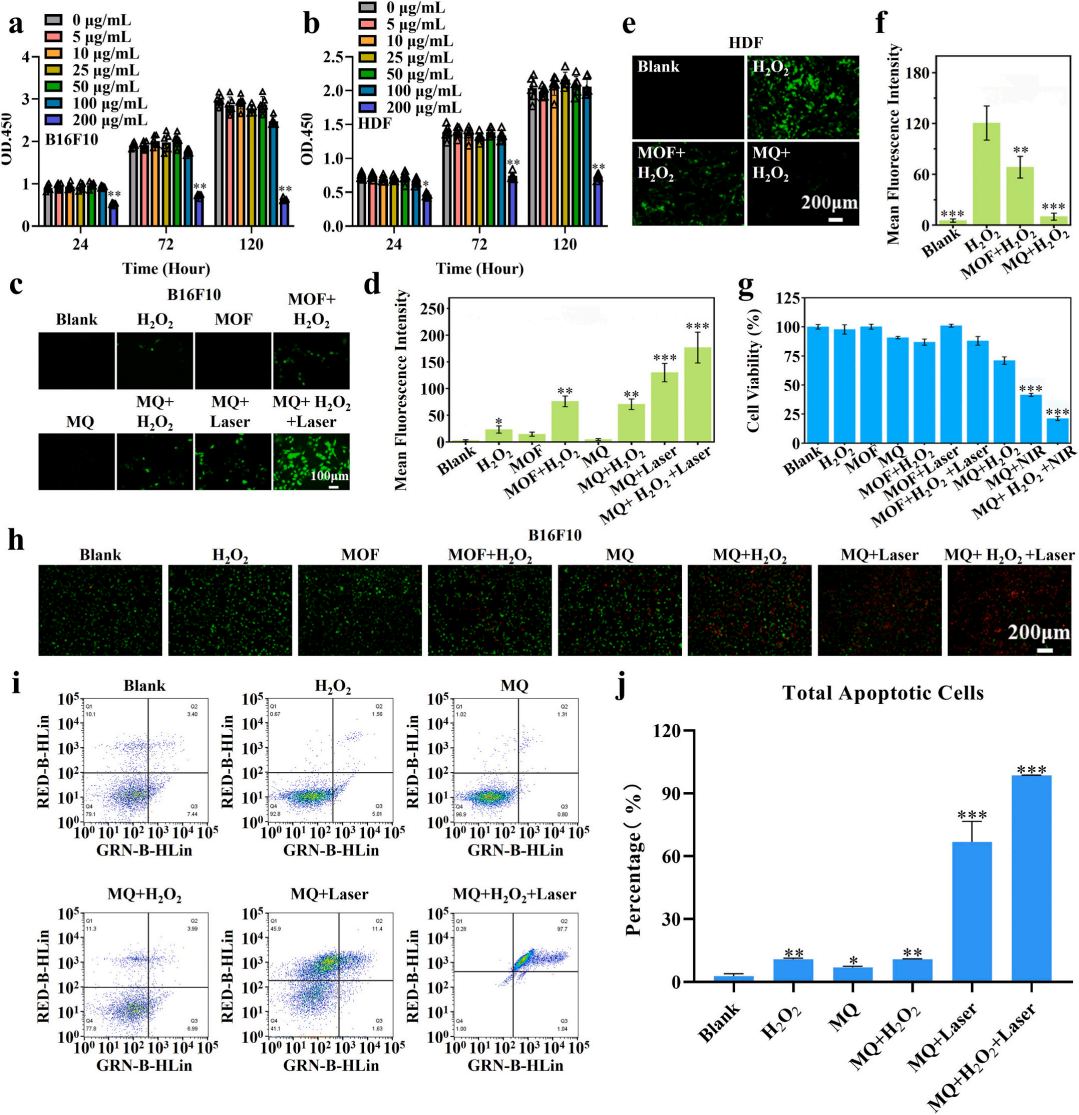
**In vitro cytocompatibility, light-controlled bidirectional ROS regulation, and triggered tumor cell apoptosis of the MQ complex.** (a, b) In vitro biocompatibility evaluation profiles showing the viability of (a) B16F10 melanoma cells (pH = 6.0) and (b) HDF fibroblasts (pH = 7.4) incubated with MQ at varying concentrations over 24, 72, and 120 h, determined by CCK-8 assay. (c) Representative CLSM fluorescence images of DCFH-DA staining illustrating intracellular ROS generation dynamics in B16F10 cells across distinct treatment groups (pH = 6.0). (d) Quantitative fluorescence intensity analysis corresponding to the intracellular ROS levels in B16F10. (e, f) Intracellular ROS scavenging capacity in HDF cells (pH = 7.4). (e) typical CLSM fluorescence micrographs and (f) corresponding quantitative fluorescence statistical histograms proving the mitigation of H_2_O_2_-induced oxidative stress by MOF and MQ in the dark. (g) Cell viability of B16F10 melanoma cells subjected to different therapeutic combinations with or without 660 nm laser irradiation (0.5 W/cm^2^, 10 min). (h) Representative Calcein-AM (live cells, green fluorescence) and PI (dead cells, red fluorescence) co-staining micrographs of B16F10 cells post-treatments. (i, j) Apoptosis pathway validation via flow cytometry: (i) typical scatter plots representing the distribution of apoptotic cell populations and (j) corresponding quantitative percentages of total apoptotic B16F10 cells across distinct intervention groups. *p < 0.05, **p < 0.01, and ***p < 0.001 indicate statistically significant differences. (For interpretation of the references to color in this figure legend, the reader is referred to the Web version of this article.)

Next, we investigated the light-controlled behavior of generation and scavenging of ROS *in vitro*. We first evaluated the biocompatibility of MQ *in vitro*. According to the actual tumor wound repair treatment, we chose melanoma cells (B16F10) and typical fibroblasts (HDF cells). To establish the optimal therapeutic window and rigorously assess the long-term biosafety of the MQ complex, the viability and proliferation dynamics of both B16F10 and HDF cells were continuously monitored over 120 h (Fig. 4a and b). Both cell lines exhibited unperturbed proliferative trajectories, statistically analogous to the control group, at MQ concentrations up to 100 μg/mL across all tested time points (24, 72, and 120 h). However, acute cytotoxicity was triggered universally at 200 μg/mL, evidenced by a significant abrogation of cellular proliferation. Based on these results, a concentration of 100 μg/mL was selected as the therapeutic concentration in further experiments.

To systematically evaluate the intracellular light-controlled ROS generation and scavenging profiles while validating that the photodynamically generated ROS escape premature self-neutralization within the therapeutic window, the redox behaviors under distinct simulated microenvironments were monitored via CLSM imaging using DCFH-DA probes. Under 660 nm near-infrared laser irradiation, B16F10 melanoma cells incubated with MQ in a weakly acidic tumor microenvironment (pH 6.0) exhibited a distinct green fluorescence surge even in the absence of exogenous H_2_O_2_ (Fig. 4c). This robust intracellular oxidative blast confirms the initiation of a dominant pro-oxidant pathway driven by Type-I electron-hole separation, demonstrating that the photogenerated reactive species successfully manifest inside the malignant cells without being instantly quenched or neutralized by the inherent reducing polyphenols of the complex. Upon the introduction of exogenous H_2_O_2_ to fully simulate the hostile tumor milieu, the quantitative fluorescence analysis revealed that the intracellular ROS abundance reached its absolute maximum in the MQ + H_2_O_2_+Laser cohort (Fig. 4d). This peak amplification underscores a highly efficient synergistic cascade between the photocatalytic radical generation and the iron-catalyzed Fenton-like reaction that continuously converts H_2_O_2_ into highly cytotoxic hydroxyl radicals to aggressively drive cell apoptosis. Conversely, when shifting to a neutral normal tissue environment (pH 7.4) under dark conditions to simulate the tissue-repair phase, the complex entirely flips its functional bias to act strictly as an antioxidant shield for HDF fibroblasts, as visualized by the dramatic minimization of H_2_O_2_-stimulated intracellular ROS storms (Fig. 4e). The corresponding quantitative fluorescence statistical histograms confirmed that the intracellular ROS abundance was successfully restored back to baseline physiological levels (Fig. 4f).

To substantiate the dynamic reversibility and microenvironment-dependency of the light-controlled redox switch, real-time intracellular ROS were monitored. In normal endothelial cells (HUVECs, pH 7.4), MQ induced a transient oxidative surge exclusively under near-infrared irradiation, while rapidly scavenging the accumulated ROS back to physiological baselines upon laser cessation (Fig. S14). Conversely, for B16F10 melanoma cells in acidic microenvironment (pH 6.0), severe oxidative stress persisted even after stop of the laser irradiation (Fig. S15). Strikingly, elevating the local pH to 7.4 efficiently quenched the intracellular ROS. These results suggest that MQ only create high intracellular ROS level in tumor environment, and low ROS level in wound healing process.

To evaluate the subsequent anti-tumor efficacy, the cytotoxicity of various therapeutic combinations on B16F10 cells was quantified using CCK-8 and live/dead cell staining assays. In the absence of laser irradiation, no significant cell inhibition was observed across the single-component control groups. However, under 660 nm laser excitation, the viability of B16F10 cells in the MQ + Laser and MQ + H_2_O_2_+Laser groups dropped sharply to 41.1% and 21.0%, respectively (Fig. 4g). Calcein-AM/PI co-staining further corroborated this trend (Fig. 4h), revealing widespread cell death exclusively in the laser-irradiated MQ cohorts. To resolve the underlying programmed cell death pathways, flow cytometry analysis was performed to monitor cell apoptosis. Consistent with the viability metrics, flow cytometry analysis showed that MQ coupled with laser intervention resulted in a pronounced shift in the cell population toward advanced apoptosis quadrants (Fig. 4i), leading to significant increase of the total apoptotic percentage as compared to the dark group and blank controls (Fig. 4j). Mechanistically, this high-performance tumor eradication is driven by the severe laser-triggered ROS burst, which inflicts immediate oxidative damage and aggressively activates the downstream mitochondria-mediated apoptotic cascade.

To elucidate the specific molecular pathways governing the light-triggered cytotoxicity, the transcriptional profiles of apoptosis and inflammation-related genes in B16F10 cells were quantified (Fig. S16). Upon laser irradiation, the MQ complex significantly upregulated the expression of core pro-apoptotic genes (Bax, Caspase-3, and p53) alongside pro-inflammatory cytokines (TNF-α, IL-6, and IL-1β). These distinct transcriptional shifts mechanistically verify that the severe oxidative burst induced by the MQ photocatalysis not only inflicts direct structural damage but also effectively eradicates malignant cells by aggressively activating the mitochondria-mediated apoptotic cascade and localized inflammatory responses.

Conversely, to validate the regenerative potential of MQ in the dark state, the gene expression profile of HDF cells under H_2_O_2_-induced oxidative stress was evaluated (Fig. S17). Treatment with MQ effectively downregulated the overexpressed pro-inflammatory genes (TNF-α, IL-1β) to physiological baseline levels. Concurrently, the transcriptional abundance of critical extracellular matrix components (Col1a1 and Col3a1) was dramatically upregulated. This dual-directional gene modulation rigorously proves that the reducing capacity of MQ simultaneously resolves the hostile inflammatory microenvironment and reinvigorates collagen synthesis, thereby orchestrating optimal conditions for accelerated wound remodeling.

### 2.5 Regulatory effects of MQ on endothelial cells *in vitro*

It is known that, under the influence of the wound microenvironment, endothelial cells (HUVECs) experience reduced cell viability due to oxidative stress, including impaired migration and angiogenic activity, which can affect skin wound healing [68]. Therefore, we investigated the protective effect of MQ on HUVECs under oxidative stress using the CCK-8 cell viability assay (Fig. S18). The results showed that MQ significantly enhanced HUVECs cell viability, possibly through scavenging H_2_O_2_ in cellular microenvironment to reduce oxidative stress. In comparison, MOF could not completely scavenge H_2_O_2_ in the oxidative stress microenvironment, so that the cell viability in the MOF + H_2_O_2_ group was lower as compared to the Blank group. Furthermore, the effect of MQ on HUVECs migration under oxidative stress was studied using a scratch assay. As shown in Fig. S19, H_2_O_2_ clearly inhibited migration of HUVECs, while MQ stimulate HUVECs migration even under oxidative stress. Additionally, the effect of MQ on angiogenic ability of HUVECs was evaluated using vascular ring formation assay. As shown in Fig. S20, MQ significantly stimulated HUVECs to form more vascular networks under oxidative stress as compared to the H_2_O_2_ group, in which vascular ring formation was inhibited by oxidative stress.

To detect the biological influence of the laser, the direct effects of the near-infrared laser (660 nm, 0.5 W/cm^2^, 10 min) were evaluated. The results demonstrated no significant effect on the viability of either B16F10 or HUVECs on 5 days under near-infrared laser (Fig. S21). Additionally, ESR spectroscopy confirmed that the laser alone did not induce intracellular ROS generation (Fig. S22). These data indicate that the applied laser does not independently influence tumor growth, cell proliferation, or oxidative stress levels.

### 2.6 Therapeutic effect of MQ on melanoma wounds *in vivo*

**Fig. 5:**
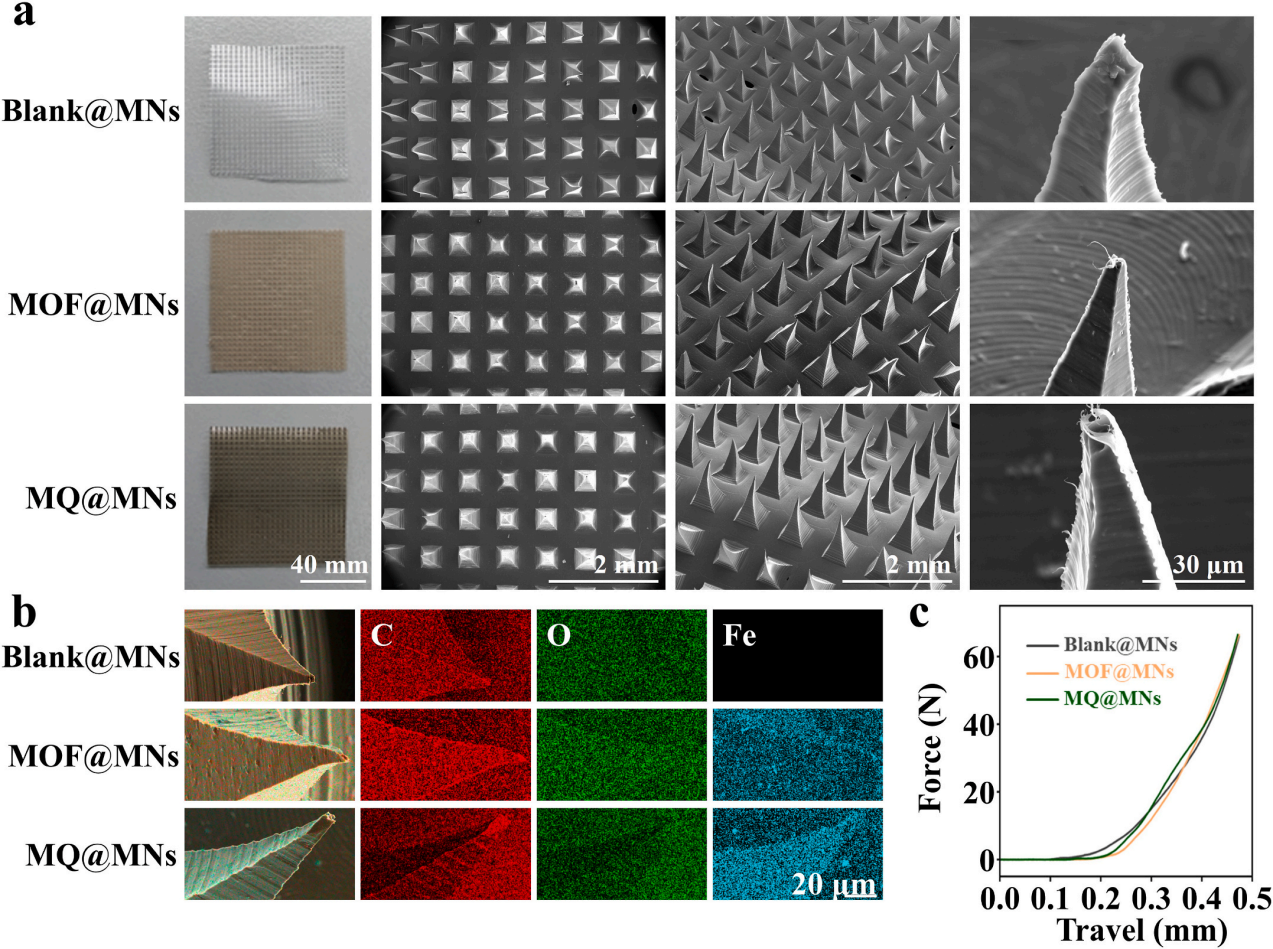
**Morphological, compositional, and mechanical characterization of the composite microneedles.** (a) Macroscopic photographs and scanning electron microscopy (SEM) images illustrating the overall array morphology, individual microneedle structures, and tip geometries of Blank@MNs, MOF@MNs, and MQ@MNs. (b) Energy-dispersive X-ray spectroscopy (EDS) elemental mapping (C, O, Fe) of the microneedles. (c) Stress-strain curves evaluating the mechanical strength and structural integrity of the respective microneedle patches.

In order to investigate the therapeutic function of MQ on tumor wound, we first prepared MQ/sodium hyaluronate composite microneedles (MQ@MNs). Scanning electron microscopy (SEM) characterization reveals that the encapsulation of the MQ complex preserves the regular, well-defined pyramidal morphology and structural fidelity of the pristine HA microneedle arrays (Fig. 5a). To further elucidate the internal compositional homogeneity and interfacial integration within the polymeric matrix, energy-dispersive X-ray spectroscopy (EDS) elemental mapping was performed (Fig. 5b). The elemental profiles demonstrate a highly continuous and uniform spatial distribution of characteristic Fe signals across both the pyramidal needle tips and the supporting backing layer, explicitly confirming that the MQ particles are homogeneously dispersed.

Axial compression measurements indicate that the incorporation of MQ does not compromise the mechanical robustness of the HA framework (Fig. 5c). The composite microneedles exhibit a critical failure force substantially exceeding the threshold required to breach the cutaneous barrier, which is mechanistically attributed to the strong hydrogen-bonding network and interlocking interfaces between the polyhydroxyl segments of Quercetin/HA and the metal nodes of the MOF.

To verify the transdermal delivery efficacy dictated by the mechanical strength of the patches, the skin penetration assay was conducted. The application of MQ@MNs generates distinct micro-channels that physically breach the stratum corneum and epidermis, reaching the superficial dermis with an approximate penetration depth of 150-200 μm (Fig. S23). This structural disruption confirms that the MQ@MNs possess sufficient mechanical robustness to overcome the cutaneous barrier, ensuring the spatial localization and deep-tissue delivery of the MQ complex required for subsequent in situ redox modulation.

Furthermore, quantitative evaluation of the *in vitro* degradation kinetics (Fig. S24) reveals a steady, time-dependent mass loss profile, achieving approximately 80% degradation at 96 h. Driven by the progressive dissolution of the hyaluronic acid matrix in physiological environments, this continuous biodegradation trajectory guarantees the sustained release of the MQ.

**Fig. 6:**
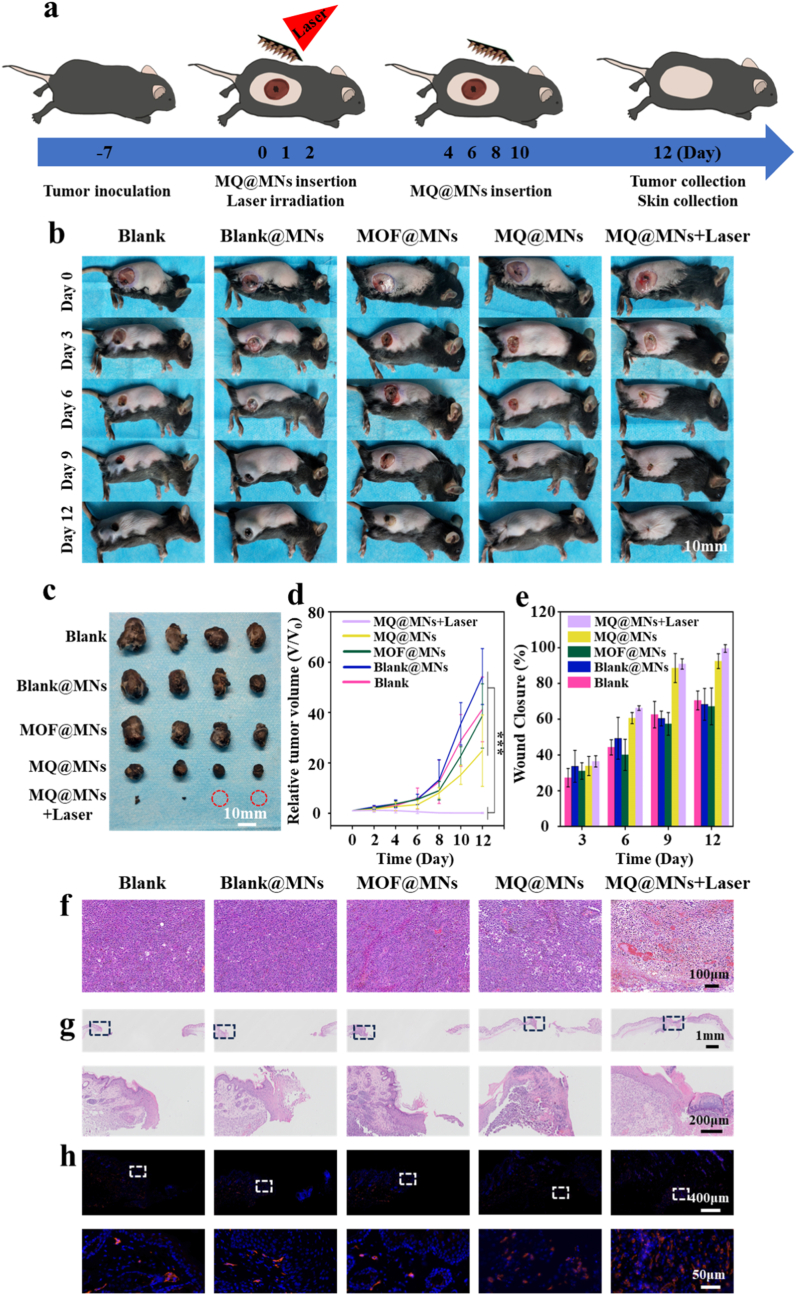
**MQ@MNs with Light-controlled Generating/Scavenging of ROS for Melanoma Wound Treatment.** (a) Schematic diagram of the treatment process in mice (laser: 660 nm, 0.5W/cm^−2^, 10min). (b) Photograph of the melanoma wound area in mice during treatment. (c) Photograph of the tumor taken out after 12 days. (d) Curves of tumor growth in B16F10 -tumor-bearing mice within 12 days. (e) Quantitative analysis of wound healing rate within 12 days. (f) H&E staining image of tumor tissue. (g) H&E staining image of skin tissue. (dashed line box showing wound area) (h) CD31 immunofluorescence staining image of skin tissue (dashed line box showing wound area) (n = 4, *p < 0.05, **p < 0.01, ***p < 0.001).

Then, we further investigated the therapeutic effect of MQ@MNs in mice with melanoma wounds [65]. In order to see the inhibitory effect of MQ on tumor re-occurrence, we established a tumor wound model by only partially excising tumor tissue during the wound creation, and treated the surgical wound with MQ@MNs with or without laser irradiation (Fig. 6a). Optical images revealed that by the 12th day, the incompletely excised melanomas in the Blank, Blank@MNs, and MOF@MNs groups continued to grow, and the wound was not completely healed. In contrast, the MQ@MNs group showed significant wound healing due to MQ wound-healing enhancing activity, and the skin wound was almost completely healed. However, the tumor continued to grow under the skin due to the lack of ROS generating activity without laser irradiation (Fig. 6b and c). Notably, compared with the Blank, Blank@MNs, and MOF@MNs groups, tumor growth in mice from the MQ@MNs group was also inhibited after treatment (Fig. 6d). This might be mainly attributed to the cytotoxic effect of MQ on tumor cells in a weakly acidic environment and its catalysis of endogenous H_2_O_2_ in the tumor to produce •OH, which inhibited the tumor to some extent. In contrast, when MQ@MNs patches were applied with Laser irradiation, tumor suppression was evident by the 3rd day, and the wound continued to heal as the treatment progressed. By the 12th day, the tumor was no longer visible, and the surgical wound was completely healed. Additionally, quantitative analysis of wound healing rates showed that on the 12th day, MQ@MNs patches achieved a wound healing rate of 90%, while the MQ@MNs + Laser group reached 100% wound healing. The other three groups showed no significant wound healing (Fig. 6e). This indicates that MQ can promote wound healing without laser irradiation, while laser irradiation of MQ@MNs patches not only inhibit the tumor growth, but is also beneficial for wound healing.

Moreover, H&E staining results of tumor tissue showed that tumor cells in the Blank, Blank@MNs, MOF@MNs, and MQ@MNs groups grew normally, while the tumor area in the MQ@MNs + Laser group exhibited significant apoptosis (Fig. 6f). This result indicates that Laser triggered ROS generation by MQ@MNs effectively induced apoptosis of tumor cells. H&E staining results of skin tissue (Fig. 6g) confirmed optical observation that tumor re-growth after surgical excision hindered wound healing, resulting in large areas of vacancy in the middle region of the sections in Blank, Blank@MNs, and MOF@MNs groups. Although MQ@MNs patches revealed activity to promote wound healing, continuous tumor proliferation also hindered complete wound healing, leaving small vacancies. In contrast, the MQ@MNs + Laser group showed complete wound healing with the growth of the dermis, epidermis, and small scars caused by surgery. It is known that vascularization is crucial for wound healing, as new blood vessels enhance delivery of sufficient oxygen, nutrients, and growth factors to the wound, and effectively promote healing. Immunofluorescence staining results (Fig. 6h) showed that on the 12th day, the MQ@MNs group and MQ@MNs + Laser group revealed significantly enhanced fluorescence intensity as compared with the Blank, Blank@MNs, and MOF@MNs groups, indicating stimulating CD31 expression, and the Laser irradiation of MQ@MNs further enhanced angiogenesis comparing to MQ@MNs without laser irradiation (Fig. S25). This result confirmed the *in vitro* observation and suggests that the promotion of angiogenesis may attribute to the activation of endothelial cells by MQ possiblly through the release of bioactive factors such as Qu and Qu-Fe chelates [66,67]. It is interesting to see that MQ@MNs + Laser group promote higher angiogenesis than MQ@MNs group, which may be attributed to the enhanced release of bioactive factors [69].

To further quantitatively evaluate the systemic in vivo biosafety of MQ@MNs, the body weight and core hematological biochemistry of the mice were systematically monitored. Over the 12-day treatment period, the body weight of the MQ@MNs group maintained high statistical consistency with the Blank group (Fig. S26). Furthermore, quantitative serum biochemical analysis at the treatment endpoint confirmed that the core indicators of hepatic detoxification (ALT, AST, Fig. S27) and renal filtration clearance (BUN, CREA, Fig. S28) strictly remained at physiological baseline levels, with no significant differences between the groups. These quantitative hematological data indicate the biocompatibility of the MQ@MNs at both molecular and tissue dimensions. Furthermore, we evaluated important organs after the treatment. The H&E staining of the internal organ tissue sections of mice (Fig. S29) revealed no significant differences between the MQ@MNs + Laser group and the Blank group, both exhibiting good cell morphology and uniform nuclear distribution. This result indicates that the material and Laser irradiation have no significant damage to the internal organs of mice.

To further evaluate the macroscopic therapeutic outcomes and explicitly delineate the physical mechanisms underlying the in vivo tumor ablation, comparative assessments against conventional high-temperature photothermal therapy (PTT) were conducted. As demonstrated in the time-dependent macroscopic photographs (Fig. S30a), the MQ group achieved an equivalent, or even superior, tumor eradication efficacy compared to the standard PTT group, notably avoiding apparent collateral burn damage to surrounding healthy tissues. This robust anti-melanoma outcome is further corroborated by the optical morphology of the completely excised tumors at the observation endpoint (Fig. S30b). To investigate the corresponding thermal dynamics contributing to this efficacy, real-time infrared thermal imaging was continuously monitored during the treatment (Fig. S30c). Upon near-infrared irradiation, the tumor site in the MQ group exhibited a highly localized and moderate heat distribution. The subsequent temporal temperature evolution trajectories mathematically confirm that the MQ group strictly plateaued at a mild hyperthermic state, which was significantly lower than the extreme peak temperatures generated by the pure PTT control (Fig. S30d and S30f). These sequentially validated metrics fundamentally prove that the tumor ablation by MQ is predominantly driven by the light-triggered photocatalytic ROS burst (PDT) synergistically potentiated by mild thermal activation, effectively preventing the non-specific thermal necrosis typically associated with traditional PTT.

### 2.7 Evaluation of light-controlled ROS generation/scavenging of MQ *in vivo*

**Fig. 7:**
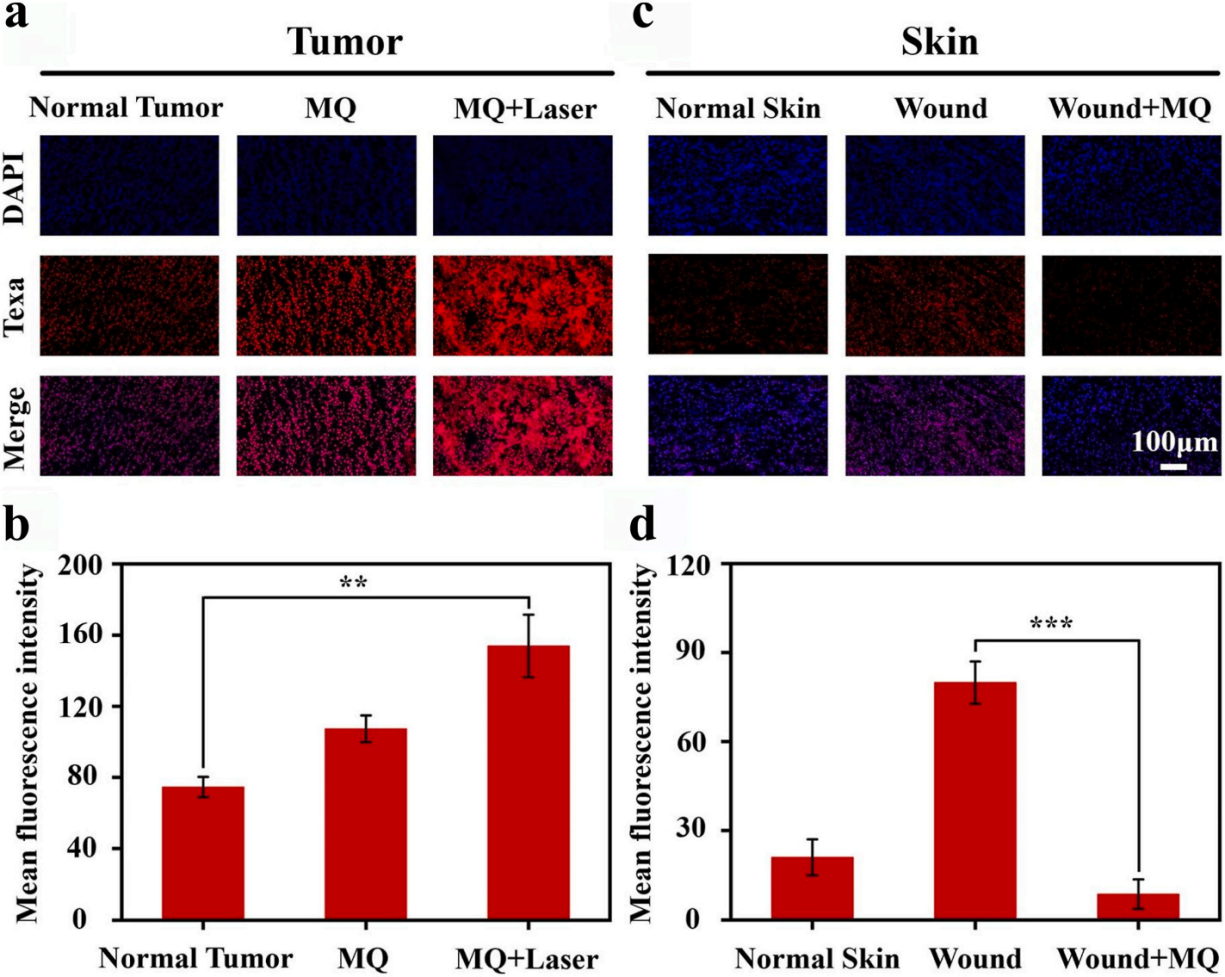
**Light-controlled Generation/Scavenging of ROS by MQ *in Vivo.*** (a, b) The CLSM images of DHE staining and quantitative fluorescence analysis of ROS generation in tumor tissue with laser irradiation. (c, d) The CLSM images of DHE staining and quantitative fluorescence analysis of ROS scavenging in wound skin tissue without laser irradiation. (Laser parameters: 660 nm, 0.5W/cm^−2^, 10min). n = 4, *p < 0.05, **p < 0.01, ***p < 0.001.

We further investigated redox dual functionality of MQ by analyzing the ROS generation and scavenging in vivo by Dihydroethidium (DHE) staining, and the distribution of ROS in tumor and skin tissue was observed under confocal laser scanning microscope (CLSM) (Fig. 7a and b). Tumor tissue exhibited ROS fluorescence due to the presence of H_2_O_2_, and this fluorescence became more pronounced after applying MQ@MNs. This is because MQ undergoes a Fenton-like reaction in the weakly acidic environment of solid tumors, converting H_2_O_2_ within the tumor tissue into •OH. Upon laser irradiation, the ROS content in the tumor further increased significantly. This is because MQ can convert H_2_O in biological tissues into •OH and O_2_ in the air into •O_2_^−^. This phenomenon indicates that MQ can synergistically generate a storm of ROS in a simulated solid tumor microenvironment, causing damage to tumor cells. Meanwhile, the analysis of ROS in skin areas revealed higher ROS fluorescence signals in tumor wound skin as compared with normal skin. In contrast, after applying MQ@MNs, the ROS fluorescence signal at the wound site significantly decreased, reaching the level of normal skin. This result suggests that MQ@MNs can effectively scavenge ROS in the wound skin area, protecting cells from oxidative stress and thereby promoting wound healing. Quantitative analysis further confirmed these observations (Fig. 7c and d).

### 2.8 Mechanism study of laser-controlled functional transition via transcriptomic analysis

**Fig. 8:**
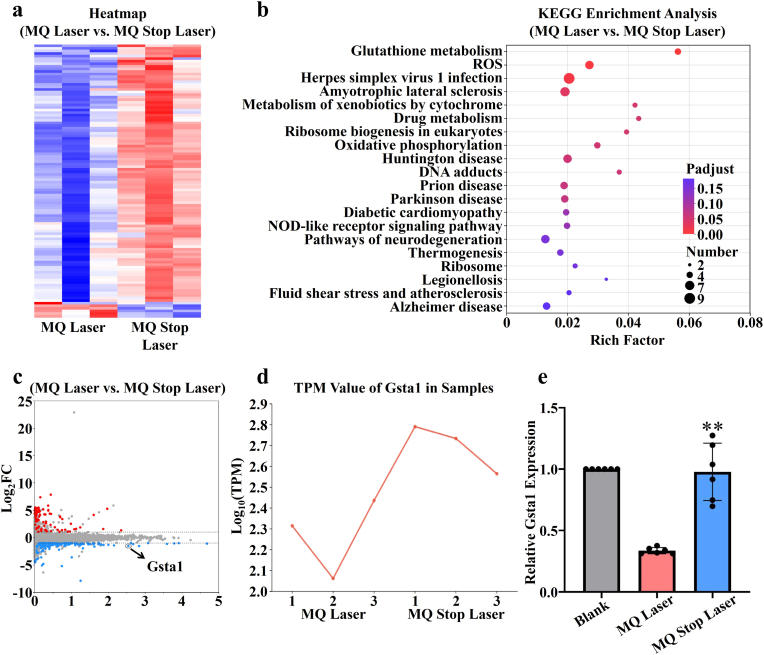
**MQ laser intervention dynamically remodels the transcriptomic profile and reversibly regulates the transcription of the target gene Gsta1.** (a) Hierarchical clustering heatmap of differentially expressed genes (DEGs) under the conditions of continuous MQ laser intervention (MQ Laser) and cessation of intervention (MQ Stop Laser). (b) KEGG pathway enrichment analysis of the DEGs. (c) Scatter plot of the gene expression distribution between the two groups. (d) Dynamic evolution trajectory of Gsta1 transcript abundance across biological replicates based on RNA-seq data. (e) Independent quantitative analysis of the relative Gsta1 expression levels. **P < 0.01.

To elucidate the molecular mechanisms underlying the light-controlled redox modulation of the MQ complex, transcriptomic profiling was conducted. The biological response is strictly mediated by photonic input, as evidenced by the distinct transcriptomic shift and global remodeling of differentially expressed genes (DEGs) between the laser irradiation (MQ Laser) and cessation (MQ Stop Laser) phases (Fig. 8a). KEGG pathway enrichment analysis demonstrates that these DEGs are significantly concentrated in Glutathione metabolism and Reactive oxygen species (ROS) pathways (Fig. 8b). This molecular signature directly corresponds to the physicochemical characteristics of MQ, wherein laser excitation triggers electron-hole separation to induce an oxidative burst, while the absence of irradiation restores its MOF-polyphenol reducing state.

Further screening of the global transcriptomic distribution (Fig. 8c) identifies Gsta1 (Glutathione S-transferase alpha 1) as a pivotal regulatory node. The transcriptional abundance of Gsta1 is markedly suppressed under continuous laser irradiation but undergoes complete reversible recovery upon laser cessation (Fig. 8d). Independent quantitative evaluation (Fig. 8e) verifies this reversible dynamic, showing that Gsta1 relative expression drops significantly during the photodynamic oxidative phase and returns to baseline levels without irradiation. Mechanistically, the transient suppression of Gsta1 impairs cellular antioxidant defense, synergizing with MQ-catalyzed ROS generation to efficiently induce tumor cell apoptosis. Conversely, the transcriptional restoration of Gsta1 upon laser cessation, coupled with the ROS-scavenging activity of the MQ complex, facilitates the alleviation of oxidative stress required for skin wound healing.

## 3 Conclusion

In this study, we synthesized a Qu modified MOF complex (MQ) with light-controlled ROS generation/scavenging function by utilizing the reducibility and facile coordination properties of Qu with Fe, which exhibits dual functionality of generation ROS under laser irradiation, and ROS scavenging without laser irradiation The synthesized MQ particles exhibit exceptional light absorption capacity and oxygen vacancies, endowing them with good photocatalytic capabilities. Notably, MQ as a metal-polyphenol complex possesses remarkable antioxidant properties. *In vitro* experiments demonstrated that MQ generated ROS under laser irradiation, effectively inhibited tumor cell growth, and scavenged ROS in the absence of laser irradiation to alleviate local oxidative stress in the wound site. Furthermore, its unique bioactivity promoted endothelial cell angiogenesis. The in vivo activity of MQ was confirmed by applying MQ/hyaluronate composite microneedles (MQ@MNs) in a mouse melanoma wound model, which effectively eliminated residual tumors and inhibit tumor recurrence by generating ROS, and simultaneously promoted skin wound healing by scavenging ROS and stimulating angiogenesis. This study suggests that the metal-polyphenol chelation based approach and polyphenol-MOF complex may have potential for development of multifunctional nanomaterials for tumor treatment and tissue regeneration.

## 4 Materials and methods

### 4.1 Materials

Iron trichloride hexahydrate (FeCl_3_·6H_2_O), terephthalic acid (H_2_BDC), quercetin dihydrate (Qu), sodium acetate (CH_3_COONa), potassium persulfate (K_2_O_8_S_2_), sodium nitrite (NaNO_2_), sodium hydroxide (NaOH) and hydrogen peroxide(H_2_O_2_) were purchased from China National Pharmaceutical Group (Shanghai, China) Co., Ltd. 3,3′,5,5′-tetramethylbenzidine (TMB), 2,2′-azinobis-(3-ethylbenzthiazoline-6-sulphonate) (ABTS), 2,2-diphenyl-1-picrylhydrazyl (DPPH), methylrosanilinium chloride (C_25_H_30_N_3_Cl·9H_2_O), and sodium hyaluronate ((C_14_H_20_NO_11_Na)_n_) were purchased from Shanghai Aladdin Biochemical Technology Co., Ltd. 5,5-dimethyl-1-pyrroline-N-oxide (DMPO) was purchased from Sigma-Aldrich Co. (Shanghai, China). Singlet Oxygen Sensor Green (SOSG) and 2,2,6,6-tetramethylpiperidine (TEMP) were obtained from Sigma-Aldrich Co. (Shanghai, China). All reagents were used as received.

### 4.2 Synthesis of MIL-101 (Fe)

The solvothermal method is used for the synthesis of MIL-101 (Fe) MOF. First, 676 mg FeCl_3_·6H_2_O was dissolved in 25 mL N-N dimethylformamide (DMF) to prepare solution A, and 1202 mg H_2_BDC was dissolved in 25 mL DMF to prepare solution B. Solution B was slowly poured into solution A with continuously stirring for 10 min, and the mixed solution was then poured into two 50 mL-polytetrafluoroethylene linings, sealed with a high-pressure reactor, and kept in a drying oven at 110 °C for 20 h. After cooling to room temperature, orange products were obtained by centrifuging at 10000 rpm for 10 min. The products were then washed with DMF three times to remove unreacted precursors, washed with ethanol three times to remove residual DMF, and soaked in 70 °C hot ethanol for 3 h and vacuumed at 60 °C for 24 h to obtain MIL-101 (Fe) MOF, which were sealed and stored for future use.

### 4.3 Synthesis and characterization of MIL-101(Fe)@Quercetin (MQ)

Firstly, 30 mg of MIL-101 (Fe) and 30 mg of Qu were dispersed in 50 mL of ethanol solution, which were subsequently dispersed uniformly in 50 mL of an ethanol solution. The mixture was then gently stirred for a continuous period of 72 h under light-protected conditions to ensure optimal reaction. Following the stirring process, the reaction solution underwent centrifugation at a high speed of 10,000 rpm for 10 min, resulting in the isolation of a black precipitate. This precipitate was meticulously washed three times, alternating between ethanol and deionized water, to eliminate any impurities. Ultimately, the purified product was subjected to freeze-drying in a specialized machine for 48 h, yielding the final compound, MIL-101(Fe)@Quercetin (MQ).

For the comprehensive analysis of MQ, a suite of characterization techniques was employed. Elemental mapping and scanning electron microscopy (SEM) images were acquired using the S- 8100 instrument (Hitachi Ltd., Japan). Transmission electron microscope (TEM) images were captured with the Hitachi-HF5000 (Hitachi Ltd., Japan). X-ray diffraction (XRD) patterns were recorded using the D/max 2550V system (Nippon Nikkei Pillar Co., Japan), covering a 2θ range from 5° to 50° to elucidate the crystalline structure of MQ. Raman spectra, revealing vibrational modes and molecular interactions, were measured with a Variable Temperature Raman Spectrometer (Renishaw, UK). The elemental composition, focusing on carbon (C), oxygen (O), and iron (Fe), was determined through X-ray photoelectron spectroscopy (XPS, US, ESCALAB 250). Electron spin resonance spectroscopy (ESR) data were obtained using the Bruker EMX1598 spectrometer, providing valuable information on unpaired electrons and magnetic properties. UV-Vis diffuse reflection spectroscopy was conducted on a UV-2600 spectrophotometer (Shimadzu Ltd., Japan) to assess the optical absorption characteristics of MQ. Laser irradiation experiments were performed utilizing a Fiber-660 laser (660 nm, Fiber Laser Technology Co., Ltd., Shenzhen), enabling precise control over excitation conditions. Atomic Force Microscopy (AFM) and Kelvin Probe Force Microscopy (KPFM) measurements were conducted using the NTEGRA system (NT-MDT, Russia) to evaluate the surface potential mapping of MQ. The hydrodynamic diameter and Zeta potential of the pristine MOF and MQ complex were measured using Nanoparticle Size and Zeta Potential Analyzer (Malvern Zetasizer Nano ZS90, UK). All samples were fully dispersed in deionized water (pH 7.0) via ultrasonication at a concentration of 100 μg/mL. The measurements were conducted at 25 °C. Each sample was measured in triplicate to ensure reproducibility. Quantitative elemental analysis of the samples was recorded using inductively coupled plasma optical emission spectrometry (ICP-OES, Varian 715-ES). Finally, optical images of cells were acquired using an inverted microscope (Leica Microsystems, Germany) and confocal laser scanning microscopy (TCS SP8, Leica, Germany).

### 4.4 Reduction performance of MQ

The ABTS detection solution was prepared as following steps: 384 mg 2,2′-azinobis-(3-ethylbenzthiazoline-6-sulphonate) (ABTS) was added to 10 mL deionized water to form reagent I, and 134 mg K_2_O_8_S_2_ was added to 10 mL deionized water as reagent II. Reagents I and II (1:1) were mixed and incubated for 12 h in dark, and diluted 10 times to form ABTS detection solution. In order to study the scavenging ability of MQ on ABTS free radicals, MQ was dispersed in deionized water at a concentration of 100 μg/mL and added to ABTS detection solution. After a 12-h incubation in dark, the absorbance value of the reacted solution at around 730 nm was measured using a UV-vis spectrophotometer (UV-2600, Shimadzu Ltd., Japan). The ABTS detection solution without adding samples was used as the Blank control, and the solution obtained by reacting the deionized water dispersion of MOF (concentration of 100 μg/mL) with the ABTS working solution is used as the positive control.

The DPPH detection solution was prepared as follows: 4 mg 2,2-diphenyl-1-picrylhydrazyl (DPPH) was added to 50 mL ethanol and sonicated for 10 min to form DPPH detection solution. Then, MQ dispersed in anhydrous ethanol at a concentration of 100 μg/mL was added to the DPPH detection solution and incubated in dark for 12 h. A UV-vis spectrophotometer was used to detect the absorbance value of the reacted solution at around 520 nm. The DPPH detection solution without adding samples served as Blank control, and the ethanol dispersion of MOF (concentration of 100 μg/mL) reacted with DPPH detection solution as the positive control group.

### 4.5 Calculation of quercetin (Qu) loading in MQ

To determine the quercetin (Qu) loading in MQ, a standard curve for the Qu ethanol solution was first established. This was achieved by leveraging the linear relationship between the concentration of Qu and its absorbance. Specifically, Qu ethanol standard solutions were prepared at concentrations of 1, 5, 10, 25, and 50 ppm. The absorbance of each solution was then measured using a UV-vis spectrophotometer, and the resulting data were subjected to linear fitting to generate the standard curve.

During the preparation of MQ, waste solution were collected, and measures were taken to ensure that the volume of the solution remained constant before and after the reaction. The volume of the entire reaction system was denoted as V_(reaction system)_. The initial and final concentrations of Qu in the reaction were represented as C_1(Qu)_ (before reaction) and C_2(Qu)_ (after reaction), respectively. The weight of the MQ product obtained was recorded as m_(MQ)_. By referring to the established standard curve, the mass of Qu consumed during the reaction, denoted as m_(Qu)_, could be accurately determined. Subsequently, the drug loading rate of Qu in MQ was calculated based on these measured values. The specific formula is as follows:

V_(C1(Qu)- C2(Qu))_ = m_(Qu)_

C2(Qu) = absorbance point in standard curve.

Drug loading rate=(m(Qu)/m(MQ))*100%

### 4.6 Comparison of restorability between Qu and MQ

The mass of Qu contained in MQ was calculated based on the drug loading rate. 1 mg MQ and the corresponding mass of Qu were dispersed in 10 mL ABTS or DPPH solutions. At different time point (0, 0.5, 2, 6, 12 and 24 h), 1 mL mixed solution was centrifuged (16000 rpm) to obtain the supernatant, then a UV-vis spectrophotometer was used to detect changes in absorbance.

### 4.7 Reactive oxygen species (ROS) generation and scavenging

3,3′,5,5′-tetramethylbenzidine (TMB) can be oxidized by •OH to form a blue oxidized state of TMB-OH. The ability of MQ to produce •OH under external stimuli is analyzed based on the colorimetric reaction. The specific operation steps are as follows: MQ (100 μg/mL) was dispersed in 2 mL sodium acetate buffer (20 mM, pH = 6.0) and added to a quartz colorimetric dish. 20 μL TMB/anhydrous ethanol (10 mg/mL) test solution was then added and mixed well, and irradiated with a 660 nm (0.5 W/cm^2^) laser for 5 min. The absorbance change was measured at 650 nm by a UV-vis spectrophotometer. The MOF group and the Blank group were used as the control group.

5,5-dimethyl-1-pyrroline-N-oxide (DMPO) was used as a capture agent for hydroxyl radicals (•OH) and superoxide anion radical (•O_2_^−^). The ability of MQ to produce •OH and •O_2_^−^ was tested using electron spin resonance spectroscopy (ESR) characterization. When DMPO captures •OH, ESR can detect 4 peaks of features (with a peak to height ratio of 1:2:2:1). When DMPO captured •O_2_^−^, ESR can detect 6 peaks of features. The specific operation steps are as follows: In an EP tube, MQ (100 μg/mL) was dispersed in sodium acetate buffer (20 mM, pH = 6.0) containing H_2_O_2_ (10 mM) or not, 10 μL of capture agent DMPO was added. The mixture was then irradiated with 660 nm (0.5 W/cm^2^) NIR laser for 5 min, and placed in the ESR instrument for detection. Mixture without irradiation and without MQ added were used as the control groups.

To evaluate the net reactive oxygen species (ROS) generation capacity and strictly verify that the light-triggered ROS generation surpass the scavenging efficiency of the material, a 3,3′,5,5′-tetramethylbenzidine (TMB) colorimetric assay was employed. Briefly, the MQ complex (100 μg/mL) was dispersed in a sodium acetate buffer (20 mM, pH 6.0) containing the TMB test solution (10 mg/mL in ethanol). To induce the active oxidative state, the test dispersion was exposed to continuous near-infrared laser irradiation (660 nm, 0.5 W/cm^2^) for 10 min. Concurrently, an identical MQ dispersion was incubated strictly in the dark for the same duration to serve as the baseline control for ROS scavenging capacity. A blank control without MQ was also maintained under identical conditions. The net oxidative efficacy was determined by quantitatively analyzing the absorbance of the oxidized TMB products at 652 nm using a UV-vis spectrophotometer.

To evaluate the potential generation of singlet oxygen (1O^2^) and definitively exclude the Type-II photocatalytic pathway, Singlet Oxygen Sensor Green (SOSG) fluorescence assay and specific ESR spin-trapping were employed. For the SOSG assay, MQ (100 μg/mL) was mixed with SOSG reagent (2.5 μM) in deionized water. The dispersion was then irradiated with a 660 nm laser (0.5 W/cm^2^) for 30 min. The fluorescence intensity was recorded using a fluorescence spectrophotometer with an excitation wavelength of 504 nm and an emission wavelength of 525 nm. For the ESR detection of ^1^O_2_, 2,2,6,6-tetramethylpiperidine (TEMP) was utilized as the specific spin trap. Briefly, MQ (100 μg/mL) was dispersed in deionized water containing TEMP (20 mM). The mixture was irradiated with a 660 nm laser (0.5 W/cm^2^) for 5 min and immediately transferred to a capillary tube for ESR measurement to monitor the characteristic 1:1:1 triplet signal of TEMPO.

A hydrogen peroxide detection kit was used to evaluate the ability of MQ to remove H_2_O_2_. The detection buffer was prepared according to the kit instructions. MQ dispersed in deionized water (100 μg/mL) was added to 10 mM H_2_O_2_ solution and incubated in dark for 6 h. 100 μL supernatant was extracted through centrifugation at 16000 rpm for 10 min 50 μL of supernatant was mixed with 50 μL detection buffer and incubated for 5 min. The absorbance was measured at around 415 nm using UV-vis spectrophotometer. H_2_O_2_ solution without adding samples was Blank control, and H_2_O_2_ solution with MOF (100 μg/mL) was used as the positive control.

To systematically evaluate the impact of the MOF:Qu stoichiometric ratio on the light-controlled redox behavior, a series of complexes were prepared with varying MOF to Qu mass ratios (8:1, 4:1, 2:1, 1:1, 1:2, 1:4, and 1:8). The H_2_O_2_ clearance efficiency of each formulation was quantified using the aforementioned hydrogen peroxide detection kit. For the light-triggered group, the mixed solutions were exposed to continuous near-infrared laser irradiation (660 nm, 0.5 W/cm^2^) for 10 min prior to centrifugation and absorbance measurement, while the dark group was processed identically without laser exposure.

In order to study the ability of MQ to reduce •ONOO^−^, the •ONOO^−^ solution were prepared as follows: First, an equal volume of NaNO_2_ solution (50 mM) and H_2_O_2_ solution (25 mM) were added to a beaker and stirred for 5 min. Then, 10 mL HCl (1 M) and 10 mL NaOH (1.5M) were sequentially added within 1 s, and stirred until the solution changed from transparent to light yellow. The entire reaction process was carried out in an ice bath. The prepared •ONOO^−^ solution was diluted until the absorbance value is approximately 1.2 at around 302 nm, which we obtain the final •ONOO^−^ working buffer. Subsequently, MQ (100 μg/mL in deionized water) was added to the •ONOO^−^ working buffer. After 30 min incubation, the absorbance values at around 302 nm were recorded using UV-vis spectrophotometer. The •ONOO^−^ working buffer without adding samples serves as Blank control, and MOF (100 μg/mL) reacted with •ONOO^−^ working buffer as the positive control.

A total SOD enzyme assay kit was used to evaluate the ability of MQ to reduce •O_2_^−^ using. According to the reagent instructions, 10 μL enzyme solution, 80 μL WST-8 solution and 200 μL SOD sample buffer were mixed to form solution I. 1 μL reaction starter solution and 39 μL SOD sample buffer were mixed to form solution II. SOD sample buffer with dispered MQ (100 μg/mL) was added to solution I, then 20 μL of solution II was added. After 30 min of incubation, the absorbance value of the reacted liquid at around 460 nm was measured with a UV-vis spectrophotometer. The solution without adding samples is used as the Blank control, and the solution added with MOF (100 μg/mL) is used as the positive control.

### 4.8 Surface potential generation by MQ after NIR irradiation

The Kelvin Probe Force Microscopy (KPFM) technique was used to characterize the surface potential of MQ. The specific operation steps are as follows: A trace amount of MQ was fully dispersed in anhydrous ethanol and dripped onto a conductive mica sheet. The mica sheet was dried on a baking machine and placed on Atomic Force Microscope (AFM, NT-MDT, Russia), irradiated with a 660 nm (0.5 W/cm^2^) laser. The change in surface potential of the MQ was measured. The MOF was used as the control group.

### 4.9 Photoresponse performance of MQ

The photoresponse performance of MQ was measured by electrochemical experiments. Fluorine-doped tin oxide (FTO) glasses with photocatalyst deposited on the surface were used as working electrode, Na_2_SO_4_ aqueous solution (0.2M) as electrolyte solution, Ag/AgCl as reference electrode, and Pt sheet as counter electrode. Before the experiment, three FTO glass plates were cleaned with alcohol and dried. Then 10 mg sample powder of Qu, MOF and MQ were dispersed in 1 mL distilled water and ultrasonically dissolved respectively. The tested samples were deposited on FTO glass sheets with a coating area of 1 cm^2^ and the working electrode was treated in the oven for 2 h at 150 °C. For balancing the activity and stability of the sample on the working electrode, 50 mL Na_2_SO_4_ aqueous solution was measured as the electrolyte solution with the concentration of 0.2 M.

A Fiber-660 (660 nm, Fiber Laser Technology Co., Ltd. ShenZhen) with the power density 0.5 W/cm^2^ as the light source was illuminated on the surface of cathode through the light window with a grating interval time of 20 s. The electrochemical workstation was ShangHai CHI 760E.

### 4.10 Fe-Qu chelates release performance of MQ in biological microenvironment

In order to measure the release of Fe^3+^ ions from MQ in the biological microenvironment, serum-free tumor culture medium (RMPI 1640, pH = 6.0) was used to simulate the weak acid environment of solid tumors, and serum-free high sugar culture medium (HDMEM, pH = 7.4) was used to simulate the wound microenvironment. First, MQ were separately mixed with HDMEM and RMPI 1640 at 1 mg/mL, and vibrated at 37 °C. Subsequently, at defined time intervals (1, 3 and 5 days), the supernatant was obtained by 0.22 μm filtration membrane. The concentrations of Fe^3+^ ions in the supernatant were determined by an inductively coupled plasma optical emission spectrometer (ICP-OES, Varian 715-ES). The dispersion of MOF (1 mg/mL) in culture medium was used as positive control.

The release of Fe-Qu chelates in the biological microenvironment in MQ was measured by specific wavelength absorbance. Considering the interference of the color of the culture medium on the test results, sodium acetate buffer (HAc-NaAc, 20 mM, pH = 6.0) was used to simulate the weak acidic environment of solid tumors, and phosphate buffered saline (PBS, pH = 7.4) was used to simulate the wound microenvironment. First, MQ were separately mixed with HAc-NaAc and PBS at 1 mg/mL, and vibrated at 37 °C. Subsequently, at defined time intervals (1, 3 and 5 days), the supernatant was obtained by high-speed centrifugation (16000 rpm/min). The concentrations of Fe-Qu chelate in the supernatant were determined by an UV-vis spectrophotometer. MOF dispersed buffer (1 mg/mL) was used as the positive control.

### 4.11 Biosafety evaluation of the excitation source

To verify the inherent safety of the laser, pristine B16F10 and HUVECs cultured in 96-well plates were directly subjected to near-infrared laser irradiation (660 nm, 0.5 W/cm^2^, 10 min) in fresh medium without the addition of any nanomaterials. After a subsequent 24 h standard incubation, cell viability was quantified via the CCK-8 assay, utilizing non-irradiated cells cultured under identical conditions as the blank control. For intracellular ROS evaluation, B16F10 cells were incubated with the specific spin trap BMPO (50 mM) and subsequently exposed to the identical laser irradiation protocol. The cell suspensions were immediately transferred to capillary tubes, and ESR spectra were recorded to monitor radical generation. Cells treated with H_2_O_2_ (100 μM) served as the positive control.

### 4.12 In vitro cytotoxicity analysis

The cell viability was evaluated by Cell Counting Kit-8 (CCK-8). Human Dermal Fibroblasts-adult (HDF-a) were cultured in Dulbecco's Modified Eagle Medium-High Glucose (HDMEM) including 10% fetal calf serum (FBS) and 0.5% penicillin/streptomycin. Mouse skin melanoma cells (B16F10) were cultured in Roswell Park Memorial Institute-1640 medium (RPMI-1640) including 10% fetal calf serum (FBS) and 0.5% penicillin/streptomycin, and the pH of the medium was controlled to simulate the weak acidity of solid tumors (pH = 6.0). Human Umbilical Vein Endothelial Cells (HUVECs) cultured in Dulbecco's Modified Eagle Medium-High Glucose (HDMEM) including 10% fetal calf serum (FBS), endothelial cell growth supplement (ECGS), 0.5% penicillin/streptomycin. To verify the biocompatibility of MQ, HDF and B16F10 cells were cultured in 96-well plates (5 × 10^3^ cells in 100 μL HDMEM and RMPI-1640 per well) in the humidified conditions with 5% CO_2_ and 37 °C. After 1 day the medium was replaced with RMPI-1640 and HDMEM with MQ at different concentrations (0, 5,10, 25, 50, 100 and 200 μg/mL) and cells were further incubated for 24, 72 and 120 h. The cells were then washed with PBS twice and incubated with fresh HDMEM and RPMI-1640 with 10% CCK8 for 2 h. A microplate reader was used to measure the absorbance of each well at the featured wavelength (450 nm).

### 4.13 In vitro ROS regulating

2′,7′-Dichlorodihydrofluorescein diacetate (DCFH-DA) was employed to qualitatively assess the intracellular reactive oxygen species (ROS) content using a fluorescence microscope. B16F10 cells were seeded in 6-well plates at a density of 4 × 10^5^ cells per well in 2 mL of RPMI-1640 medium (pH = 6.0). The cells were then incubated under humidified conditions with 5% CO_2_ at 37 °C for 24 h. After incubation, the cells were washed twice with phosphate-buffered saline (PBS). Subsequently, fresh RPMI-1640 medium containing different treatments was added to each well: blank control (Blank), hydrogen peroxide (H_2_O_2_, 100 μM), metal-organic framework (MOF, 100 μg/mL), a combination of MOF and H_2_O_2_ (MOF + H_2_O_2_, 100 μg/mL and 100 μM, respectively), MQ (100 μg/mL), a combination of MQ and H_2_O_2_ (MQ + H_2_O_2_, 100 μg/mL and 100 μM, respectively), MQ combined with near-infrared (NIR) irradiation (MQ + NIR, 100 μg/mL, 660 nm, 0.5 W/cm^2^, 10 min), and MQ combined with both H_2_O_2_ and NIR irradiation (MQ + H_2_O_2_ + NIR, 100 μg/mL, 100 μM, 660 nm, 0.5 W/cm^2^, 10 min). The cells were further incubated for 4 h. After that, the spent culture media were replaced with 2 mL of RPMI-1640 medium containing DCFH-DA (10 × 10^−6^ M), and the cells were incubated in the dark for 0.5 h. Finally, the cells were washed three times with PBS, and ROS generation was measured using fluorescence microscopy.

Human dermal fibroblast (HDF) cells were seeded in 6-well plates at a density of 4 × 10^5^ cells per well in 100 μL of Dulbecco's modified Eagle's medium (DMEM) (pH = 7.4). The cells were incubated under humidified conditions with 5% CO_2_ at 37 °C for 24 h. After incubation, the cells were washed twice with PBS and then replaced with fresh DMEM containing different treatments: blank control (Blank), H_2_O_2_ (500 μM), a combination of MOF and H_2_O_2_ (MOF + H_2_O_2_, 100 μg/mL and 500 μM, respectively), and a combination of MQ and H_2_O_2_ (MQ + H_2_O_2_, 100 μg/mL and 500 μM, respectively). The cells were incubated for 4 h. Subsequently, the spent culture media were replaced with 2 mL of DMEM containing DCFH-DA (10 × 10^−6^ M), and the cells were incubated in the dark for 0.5 h. Finally, the cells were washed three times with PBS, and ROS scavenging was measured using fluorescence microscopy.

To systematically evaluate the dynamic and reversible intracellular ROS modulation orchestrated by the MQ complex, real-time tracking of intracellular ROS level was conducted utilizing the DCFH-DA fluorescent probe. HUVECs and B16F10 cells were seeded into confocal dishes at a density of 2 × 10^5^ cells/well and cultured for 24 h. For the normal physiological microenvironment model, HUVECs were incubated with the MQ complex (100 μg/mL) in standard DMEM (pH 7.4) and stained with DCFH-DA (10 μM). The cells were sequentially subjected to a precisely controlled dark-light-dark operational cycle: an initial dark baseline observation (Laser Off), followed by continuous near-infrared irradiation (660 nm, 0.5 W/cm^2^) for 10 min (Laser On), and an immediate post-irradiation dark incubation for an additional 10 min (Laser Off). To simulate the functional transition from a tumor ablation phase to a wound healing phase, B16F10 melanoma cells were initially incubated with MQ (100 μg/mL) in a weakly acidic RPMI-1640 medium (pH 6.0) and subjected to identical dark baseline and 10 min laser irradiation protocols. Immediately following the irradiation cessation, the acidic medium was rapidly aspirated and replaced with fresh, neutral RPMI-1640 medium (pH 7.4) to simulate the microenvironmental pH shift, followed by a 10 min incubation strictly in the dark. At each designated temporal node, real-time intracellular fluorescence profiles were captured using a confocal laser scanning microscope (CLSM, Leica TCS SP8, Germany). The corresponding mean fluorescence intensities across all groups were quantitatively determined using ImageJ software.

### 4.14 In vitro B16F10 therapy

B16F10 was cultured in 96-well plates (5 × 10^3^ cells in 100 μL RPMI-1640 per well, pH = 6.0) in the humidified conditions with 5% CO_2_ and 37 °C for 24h. Subsequently, these old culture mediums were washed by PBS twice and replaced by fresh RPMI-1640 containing MQ incubated for 12h, then the therapy groups included the following: Blank, H_2_O_2_ (100 μM), MOF (100 μg/mL), MQ (100 μg/mL), MOF + H_2_O_2_ (100 μg/mL, 100 μM), MQ + H_2_O_2_ (100 μg/mL, 100 μM), MOF + NIR (100 μg/mL, 660 nm, 0.5W/cm2, 10min), MQ + NIR (100 μg/mL, 660 nm, 0.5W/cm^2^, 10min), MOF + H_2_O_2_+NIR (100 μg/mL, 100 μM, 660 nm, 0.5W/cm^2^, 10 min), MQ + H_2_O_2_+NIR (100 μg/mL, 100 μM, 660 nm, 0.5W/cm^2^, 10min). Finally, washed these 96-well plates twice with PBS and took fresh RPMI-1640 including 10% CCK-8 incubated for 2h, and then a microplate reader was used to measure the absorbance of each well at the featured wavelength (450 nm).

To further confirm the effects of different tumor therapy *in vitro*, a live-dead cell staining analysis was performed using fluorescence microscopy to measure the distribution of live and dead cells in the same environment. Briefly, B16F10 tumor cells were cultured in 24-well plates (1 × 10^4^ cells in 0.5 mL RPMI-1640 per well,pH = 6.0) the humidified conditions with 5% CO2 and 37 °C for 24h. Subsequently, these old culture mediums were washed by PBS twice and replaced by fresh RPMI-1640 containing MQ incubated for another 12h, then the therapy groups included the following: Blank, H_2_O_2_ (100 μM), MOF (100 μg/mL), MOF + H_2_O_2_ (100 μg/mL, 100 μM), MQ (100 μg/mL), MQ + H_2_O_2_ (100 μg/mL, 100 μM), MQ + NIR (100 μg/mL, 660 nm, 0.5W/cm^2^, 10min), MQ + H_2_O_2_+NIR (100 μg/mL, 100 μM, 660 nm, 0.5W/cm^2^, 10min).

Afterwards, washed these 6-well plates twice with PBS and replaced with binding buffer (0.5 mL per well) including AM (4 × 10^−6^ M) and PI (6 × 10^−6^ M) for 15 min in dark. Finally, washed with PBS and measured by fluorescence microscopy. The green fluorescence (λ_ex_ = 490 nm, λ_em_ = 515 nm) and red fluorescence (λ_ex_ = 530 nm, λ_em_ = 580 nm) were represented the live and dead cells respectively.

### 4.15 In vitro HUVECs cell proliferation

HUVECs were cultured in 96-well plates (800 cells in 100 μL ECM per well) in the humidified conditions with 5% CO_2_ and 37 °C for 24h. Subsequently, the spent culture media were aspirated, and the cells were washed twice with phosphate-buffered saline (PBS). Fresh epithelial cell medium (ECM) containing MQ was then added, and the cultures were incubated for 1, 3, or 5 days, with medium replacement every 48 h. The therapy groups included the following: Blank, H_2_O_2_ (100 μM), MOF (100 μg/mL), MQ (100 μg/mL), MOF + H_2_O_2_ (100 μg/mL, 100 μM), MQ + H_2_O_2_ (100 μg/mL, 100 μM). Finally, washed these 96-well plates twice with PBS and took fresh ECM including 10% CCK-8 incubated for 2h, and then a microplate reader was used to measure the absorbance of each well at the featured wavelength (450 nm).

### 4.16 In vitro HUVECs cell migration

HUVECs were carefully seeded into 6-well plates and cultured until they attained approximately 80% confluency. Subsequently, a precise scratch was made in the cell monolayer within each well using a 1 mL plastic tip, followed by a thorough wash with phosphate-buffered saline (PBS). Initial optical images of the scratches were captured at the 0-h time point using an inverted microscope. The culture medium was then replaced with fresh endothelial cell medium (ECM) devoid of fetal bovine serum (FBS) but containing MQ. This setup was facilitated within a 6-well plate Transwell chamber, which was positioned atop the 6-well plates and incubated for a duration of 24 h. The experimental groups were meticulously designed to include: Blank, H_2_O_2_ (100 μM), MOF (100 μg/mL), MQ (100 μg/mL), MOF + H_2_O_2_ (100 μg/mL, 100 μM), MQ + H_2_O_2_ (100 μg/mL, 100 μM). Following the incubation period, the 6-well plates were washed twice with PBS, fixed with 4% paraformaldehyde for 20 min, and subsequently stained with 0.1% crystal violet. Optical images of the scratches were then captured again at the 24-h mark, utilizing the same inverted microscope for consistency. To quantitatively assess cell migration, the optical images obtained were analyzed using ImageJ software (National Institutes of Health, USA). The migration ratio was calculated based on the following formula: Migration ratio (%) = (A_0_ - A_n_)/A_0_ × 100%, where A_0_ denotes the scratch area at the 0-h time point, and A_n_ represents the scratch area at the 24-h time point.

### 4.17 In vitro tube formation evaluation

Tube formation assay was conducted using ECMatix™ (Millipore, cat. no. ECM625) according to the manufacturer's instructions. First, 24-well plates were evenly coated with ECMatix™, and HUVECs (1 × 10^5^ cells per well) were cultured on ECMatrix™. And fresh ECM containing MQ in a 24-well plate Transwell chamber placed on 24-well plates. The therapy groups included the following: Blank, H_2_O_2_ (100 μM), MOF (100 μg/mL), MQ (100 μg/mL), MOF + H_2_O_2_ (100 μg/mL, 100 μM), MQ + H_2_O_2_ (100 μg/mL, 100 μM). After 6 h, cells were fixed by 4% paraformaldehyde for 20 min, and the angiogenesis was recorded using an inverted light microscope. Finally, the number of junctions formed by HUVECs was quantified using ImageJ software (NIH, USA).

### 4.18 Transcriptomic analysis and RT-qPCR verification

For transcriptomic analysis, HUVECs were treated with MQ under laser irradiation (MQ Laser group) or after laser cessation (MQ Stop Laser group). Total RNA was extracted using TRIzol reagent (Invitrogen) according to the manufacturer's instructions. The RNA quality was assessed, and libraries were sequenced on an Illumina Novaseq platform. Differentially expressed genes (DEGs) were identified with |log2FC| > 1 and P < 0.05. KEGG pathway enrichment analysis was performed to identify the biological pathways. For RT-qPCR, cDNA was synthesized using a PrimeScript™ RT Master Mix. The relative expression of gene was calculated using the 2^−ΔΔCt^ method, with GAPDH as the internal control. The primer sequences were show in Table S2.

### 4.19 Preparation of composite microneedles

MQ@MNs were prepared using a polydimethylsiloxane (PDMS) mold (microneedle array: 100*100 mm). First, 3 mg MQ was ultrasonically dispersed in 30 mL deionized water. Then, 0.45 g sodium hyaluronate was added to the above dispersion, and the mixture was stirred continuously for 24 h until the sodium hyaluronate was fully dissolved. The mixture was then poured onto the PDMS mold and spread evenly. The mold was transferred to a vacuum drying oven and kept in vacuum for 2 h, then transferred to a light-proof dry place and left to stand for 1 week to obtain a soluble composite microneedle patch (MQ@MNs). The prepared microneedle samples were observed using a scanning electron microscope (S- 8100, Hitachi, Japan). To analyze the elemental composition and internal structural homogeneity of the MNs, Energy Dispersive X-ray Spectroscopy (EDS, S- 8100, Hitachi, Japan) elemental mapping was further performed.

### 4.20 In vitro biodegradation of microneedles

The degradation behavior of the MQ@MNs was evaluated by continuously monitoring their mass loss. Briefly, the initial dry weight of the microneedle patches was accurately recorded (W_0_). The patches were subsequently immersed in phosphate-buffered saline (PBS, pH 7.4) and incubated dynamically at 37 °C. At predetermined time intervals (6, 12, 24, 48, 72, and 96 h), the remaining patches were carefully retrieved, gently washed with deionized water to remove residual salts, lyophilized, and reweighed (W_t_). The biodegradation rate was calculated according to the formula: Biodegradation Rate (%) = (W_0_ - W_t_)/W_0_ * 100%.

### 4.21 Skin penetration assay

To evaluate the transdermal penetration capability of the microneedles, freshly excised normal dorsal skin from C57 mice was employed. The MQ@MNs patch was firmly pressed onto the epidermal side of the skin for 5 min. Subsequently, the treated skin tissues were immediately fixed in 4% paraformaldehyde, embedded in paraffin, and sectioned vertically across the punctured area. The tissue sections were then stained with H&E and observed under an optical microscope to visualize the formation and depth of the micro-channels. Normal skin without patch application was processed identically as a control.

### 4.22 In vivo tumor therapy

To establish a B16F10 cancer mouse model, 6-week-old female C57 mice were purchased from the Shanghai Laboratory Animal Center, Chinese Academy of Sciences (SLACAS). All animal experimental procedures were conducted in accordance with the Animal Research and Ethics Committee of Wenzhou Institute of University of Chinese Academy of Sciences (WIUCAS24030506). To evaluate the comprehensive in vivo therapeutic effect of MQ@MNs on melanoma wounds, the entire modeling process was as follows: 30 mice were randomly divided into 5 groups (n = 6), including Blank, Blank@MNs, MOF@MNs, MQ@MNs, and MQ@MNs + NIR (660 nm,0.5 W/cm2, 10 min). A suspension of 1 × 10^6^ B16F10 cells in 100 μL of RMPI-1640 medium without fetal bovine serum (FBS) was injected subcutaneously into the right flank of each mouse. When the tumor volume reached approximately 50 mm^3^, a full-thickness circular wound with a diameter of 10 mm was surgically created at the tumor site, and part of the tumor tissue was partially resected, leaving 1 mm^3^ of tumor tissue intact. Different MNs (square-shaped with a side length of 10 mm) were applied to cover and penetrate the wound area under pressure, and then secured to the wound with a transparent film dressing. The MNs were replaced every two days, and the tumor volume of the mice was measured. The tumor volume (V) was calculated using the following formula: V (mm^3^) = L × W^2^/2 (where L and W represent the longest and shortest diameters of the tumor, respectively). The mice were photographed every 3 days to record changes in the melanoma wounds. Throughout the entire treatment process, the survival rate of mice in all groups was 100%. The wound area was measured using ImageJ software (National Institutes of Health, USA). The wound healing rate was calculated using the following formula: Wound healing rate (%) = (S_0_ - S_x_)/S_0_ × 100%, where S_0_ is the wound area on day 0, and S_x_ is the wound area on day X (X = 0, 3, 6, 9, 12).

To meticulously decouple the therapeutic contributions of photodynamic therapy (PDT) and photothermal therapy (PTT), the melanoma-bearing mice were randomly assigned into specific treatment cohorts: Blank@MNs, MQ@MNs + PDT, and MQ@MNs + PTT. Following the application of the microneedle patches, the MQ@MNs + PDT group was subjected to mild near-infrared laser irradiation (660 nm, 0.5 W/cm^2^) to primarily trigger the ROS photocatalytic burst. Conversely, the MQ@MNs + PTT group was irradiated with an elevated laser power density (660 nm, 1.5 W/cm^2^) to deliberately induce conventional high-temperature thermal ablation.

To quantitatively evaluate the real-time thermal dynamics and strictly confirm the temperature differentials between the PDT and PTT modalities during in vivo interventions, an infrared thermal imaging camera (FLIR Systems, USA) was strategically employed. The spatial thermal distribution maps and corresponding temporal temperature evolution trajectories at the targeted tumor regions were continuously recorded and extracted throughout the entire laser irradiation duration.

### 4.23 Tissue section staining

On the 12th day after the surgery, the mice were euthanized. Subsequently, tumor tissues, skin samples surrounding the tumors of the mice were collected. These tissue samples were then fixed using 4% paraformaldehyde. The fixed tissues were embedded in paraffin and sliced into sections with a thickness of 5 μm.

For the tissue sections of tumor and skin around the tumor, the Hematoxylin-Eosin (H&E) staining method was employed to visually analyze the tumor formation status and the skin healing condition. Immunohistochemical staining with CD31 was carried out on the tissue sections of skin around the tumor to conduct an in-depth analysis of the angiogenesis in the skin at the wound site.

### 4.24 ROS evaluation in tumor tissues and skin samples surrounding tumors

Mice inoculated with B16F10 tumor cells were selected, with the requirement that the tumor volume reached 500 mm^3^. A surgical wound with a diameter of 10 mm was created above the tumor, and the composite microneedle patch was placed on the wound. After laser irradiation for 10 min, samples were taken from the tumor and the skin area around the tumor, and the samples were placed on dry ice. Subsequently, frozen sections were immediately prepared and stained with a dihydroethidium probe. The distribution of reactive oxygen species (ROS) was observed under a confocal laser scanning microscope (Leica TCS SP8, Germany). The fluorescence intensity was calculated using ImageJ software.

### 4.25 In vivo biocompatibility evaluation

To comprehensively evaluate the in vivo systemic biocompatibility of the MQ@MNs, normal mice were transdermally treated with the microneedle patches for a continuous 12-day period. Throughout this duration, the dynamic body weight changes of the mice across all cohorts were recorded every 3 days. Prior to euthanasia at the therapeutic endpoint (day 12), standard blood samples were collected via the retro-orbital venous plexus. The whole blood was allowed to clot at room temperature for 2 h and subsequently centrifuged at 3000 rpm for 10 min to isolate the serum. Standardized quantitative determinations of serum alanine aminotransferase (ALT), aspartate aminotransferase (AST), blood urea nitrogen (BUN), and serum creatinine (CREA) levels were performed utilizing a fully automated biochemical analyzer and corresponding assay kits to strictly evaluate potential hepatic and renal dysfunction. Immediately following blood collection, the mice were euthanized, and major organs (heart, liver, spleen, lungs, and kidneys) were excised, fixed in 4% paraformaldehyde, and subjected to standard hematoxylin-eosin (H&E) staining for systematic histological analysis.

### 4.26 Statistical analysis

All results were measured and expressed as mean ± standard deviation (SD). Statistically significant differences between groups were analyzed by Student's t-test with one-way analysis of variance. (*p < 0.05, **p < 0.01, ***p < 0.001).

## Funding

This research was supported by the 10.13039/501100012166National Key Research and Development Program of China (2024YFA1108403), the 10.13039/501100001809National Natural Science Foundation of China (32301096, 32571537), the 10.13039/501100012226Fundamental Research Funds for the Central Universities (2232026D43) and Yunnan Provincial Major Science and Technology Project (202402AA310058).

## CRediT authorship contribution statement

**Yuze Xu:** Project administration, Writing – original draft, Writing – review & editing. **Lan Chang:** Formal analysis, Investigation, Writing – original draft, Writing – review & editing. **Zhaowenbin Zhang:** Funding acquisition, Investigation, Project administration, Writing – original draft, Writing – review & editing. **Jiang Chang:** Funding acquisition, Investigation, Writing – original draft, Writing – review & editing.

## Declaration of competing interest

The authors declare no conflict of interest.

## Data Availability

Data will be made available on request.

## References

[1] Elsner P. (2021). Metastasizing malignant melanoma following laser therapy of a pigmented mole. Aktuelle Dermatol..

[2] Weyers W. (2019). "Personalized Excision" of malignant melanoma-need for a paradigm shift in the beginning era of personalized medicine. Am. J. Dermatopathol..

[3] Bello D.M. (2019). Indications for the surgical resection of stage IV disease. J. Surg. Oncol..

[4] Ma H., Zhou Q., Chang J., Wu C. (2019). Grape seed-inspired smart hydrogel scaffolds for melanoma therapy and wound healing. ACS Nano.

[5] Wu Z., Zhuang H., Ma B., Xiao Y., Koc B., Zhu Y., Wu C. (2021). Manganese-doped calcium silicate nanowire composite hydrogels for melanoma treatment and wound healing. Research 2021.

[6] Shan Y., Tan B., Zhang M., Xie X., Liao J. (2022). Restorative biodegradable two-layered hybrid microneedles for melanoma photothermal/chemo co-therapy and wound healing. J. Nanobiotechnol..

[7] Eggermont A.M.M., Kirkwood J.M. (2004). Re-evaluating the role of dacarbazine in metastatic melanoma: what have we learned in 30 years?. Eur. J. Cancer.

[8] Teimouri F., Nikfar S., Abdollahi M. (2013). Efficacy and side effects of dacarbazine in comparison with temozolomide in the treatment of malignant melanoma: a meta-analysis consisting of 1314 patients. Melanoma Res..

[9] Guo Y.Q., Ding Y., Li D.D., Li J.J., Peng R.Q., Wen X.Z., Zhang X., Zhang X.S. (2015). Efficacy and safety of nab-paclitaxel combined with carboplatin in Chinese patients with melanoma. Med. Oncol..

[10] Sanchez M.C., Lancel S., Boulanger E., Neviere R. (2018). Targeting oxidative stress and mitochondrial dysfunction in the treatment of impaired wound healing: a systematic review. Antioxidants.

[11] Wu K.K., Wu X.X., Guo J.S., Jiao Y.P., Zhou C.R. (2021). Facile polyphenol-europium assembly enabled functional poly(l-lactic acid) nanofiber mats with enhanced antioxidation and angiogenesis for accelerated wound healing. Adv. Healthcare Mater..

[12] Correia J.H., Rodrigues J.A., Pimenta S., Dong T., Yang Z.C. (2021). Photodynamic therapy review: principles, photosensitizers, applications, and future directions. Pharmaceutics.

[13] Gunaydin G., Gedik M.E., Ayan S. (2021). Photodynamic therapy-current limitations and novel approaches. Front. Chem..

[14] Zhang Z.W.B., Dai Q.X., Zhang Y., Zhuang H., Wang E.D., Xu Q., Ma L.L., Wu C.T., Huan Z.G., Guo F., Chang J. (2020). Design of a multifunctional biomaterial inspired by ancient Chinese medicine for hair regeneration in burned skin. ACS Appl. Mater. Interfaces.

[15] Sun M., Wang L., Zhuo Y., Xu S., Liu H., Jiang X., Lu Z., Wang X., Wang Y., Yue G., Feng B., Rao H., Wu D. (2024). Multi-enzyme activity of MIL-101 (Fe)-Derived Cascade nano-enzymes for antitumor and antimicrobial therapy. Small.

[16] Wu C., Xu D., Ge M., Luo J., Chen L., Chen Z., You Y., Zhu Y.-x., Lin H., Shi J. (2022). Blocking glutathione regeneration: inorganic NADPH oxidase nanozyme catalyst potentiates tumoral ferroptosis. Nano Today.

[17] Bao C., Wang H., Wang C., Zhang X., Zhao X., Dong C.-L., Huang Y.-C., Chen S., Guo P., She X., Sun Y., Yang D. (2023). Cooperation of oxygen vacancy and FeIII/FeII sites in H2-reduced Fe-MIL-101 for enhanced Fenton-like degradation of organic pollutants. J. Hazard Mater..

[18] Peng S., Li R., Rao Y., Huang Y., Zhao Y., Xiong M., Cao J., Lee S. (2022). Tuning the unsaturated iron sites in MIL-101(Fe) nanoparticles for reactive oxygen species-mediated bacterial inactivation in the dark. Appl. Catal. B Environ..

[19] Guo Y., Feng C., Qiao S., Wang S., Chen T., Zhang L., Zhao Y., Wang J. (2020). Magnetic Fe3O4-encapsulated VAN@MIL-101(Fe) with mixed-valence sites and mesoporous structures as efficient bifunctional water splitting photocatalysts. Nanoscale.

[20] Hu X., Zhang B., Zhang M., Liang W., Hong B., Ma Z., Sheng J., Liu T., Yang S., Liang Z. (2024). An artificial metabzyme for tumour-cell-specific metabolic therapy. Nat. Nanotechnol..

[21] Zhao L.-P., Chen S.-Y., Zheng R.-R., Rao X.-N., Kong R.-J., Huang C.-Y., Liu Y.-B., Tang Y., Cheng H., Li S.-Y. (2022). Photodynamic therapy initiated ferrotherapy of self-delivery nanomedicine to amplify lipid peroxidation via GPX4 inactivation. ACS Appl. Mater. Interfaces.

[22] Zhang Z., Li W., Chang D., Wei Z., Wang E., Yu J., Xu Y., Que Y., Chen Y., Fan C., Ma B., Zhou Y., Huan Z., Yang C., Guo F., Chang J. (2023). A combination therapy for androgenic alopecia based on quercetin and zinc/copper dual-doped mesoporous silica nanocomposite microneedle patch. Bioact. Mater..

[23] Geng H., Li Z., Li Z., Zhang Y., Gao Z., Sun L., Li X., Cui J., Ni S., Hao J. (2023). Restoring neuronal iron homeostasis revitalizes neurogenesis after spinal cord injury. Proc. Natl. Acad. Sci. U. S. A.

[24] (2024). Breaking-down tumoral physical barrier by remotely unwrapping metal-polyphenol-packaged hyaluronidase for optimizing photothermal/photodynamic therapy-induced immune response. Adv. Mater..

[25] Wang Z., Guo Y., Fan Y., Chen J., Wang H., Shen M., Shi X. (2022). Metal-phenolic-network-coated dendrimer-drug conjugates for tumor MR imaging and chemo/chemodynamic therapy via amplification of endoplasmic reticulum stress. Adv. Mater..

[26] Zhang H., Zhang J., Liu B., Xiao J., Stuart M.A.C., Hou G., Zhang H., Liang S., Li Z., Wang Q., Chen S., Li P., Li X., Li Y. (2024). Natural phenolic-metal framework strengthened mesona chinensis polysaccharides microgels for improved viability of probiotics to alleviate the liver injury and gut microbiota dysbiosis. Adv. Funct. Mater..

[27] Wen M., Wang T., Li N., Wu Y., Zhang L., Xue Y., Shang L. (2024). Polyphenol-copper derived self-cascade nanozyme hydrogel in boosting oxygenation and robust revascularization for tissue regeneration. Adv. Funct. Mater..

[28] Li Y., Miao Y., Yang L., Zhao Y., Wu K., Lu Z., Hu Z., Guo J. (2022). Recent advances in the development and antimicrobial applications of metal-phenolic networks. Adv. Sci..

[29] Wu D., Zhou B., Li J., Wang X., Li B., Liang H. (2022). Coordination-driven metal-polyphenolic nanoparticles toward effective anticancer therapy. Adv. Healthcare Mater..

[30] Xu W., Lin Z., Pan S., Chen J., Wang T., Cortez-Jugo C., Caruso F. (2023). Direct assembly of metal-phenolic network nanoparticles for biomedical applications. Angew. Chem. Int. Ed..

[31] Xie L., Li J., Wang G., Sang W., Xu M., Li W., Yan J., Li B., Zhang Z., Zhao Q., Yuan Z., Fan Q., Dai Y. (2022). Phototheranostic metal-phenolic networks with antiexosomal PD-L1 enhanced ferroptosis for synergistic immunotherapy. J. Am. Chem. Soc..

[32] Zhang P., Qiao Y., Zhu L., Qin M., Li Q., Liu C., Xu Y., Zhang X., Gan Z., Hou Y. (2023). Nanoprobe based on biominerals in protein Corona for dual-modality MR imaging and therapy of tumors. ACS Nano.

[33] Cui Y., Niu Q., Hu Z., He H., Wei X., Wang M., Chen X.-L., Wang X. (2025). Multifunctional nanozyme-hydrogel system in bacterial-infected wounds. Cell Biomaterials.

[34] Zang Y., Zhang W., Wang W., Zhao X., Wang X. (2026). TiO/CIP/SilMA hydrogels promote the repair of infected wounds by interfering with bacterial signaling and energy metabolism. Cell Biomaterials.

[35] Wang W., Gui H., Wei X., Liu H., Wang X. (2025). Cuproptosis to cuproptosis‐like: therapeutic strategies for bacterial infection. Adv. Mater..

[36] Ma X.Y., Ren X.L., Guo X.D., Fu C.H., Wu Q., Tan L.F., Li H.B., Zhang W., Chen X.D., Zhong H.S., Meng X.W. (2019). Multifunctional iron-based metal - Organic framework as biodegradable nanozyme for microwave enhancing dynamic therapy. Biomaterials.

[37] Wei Z.W., Wang L.Y., Tang C.Q., Chen S.Q., Wang Z.J., Wang Y.L., Bao J.X., Xie Y., Zhao W.F., Su B.H., Zhao C.S. (2020). Metal-phenolic networks nanoplatform to mimic antioxidant defense system for broad-spectrum radical eliminating and endotoxemia treatment. Adv. Funct. Mater..

[38] Liang Y., Hou D.Y., Ni Z., Cao M.J., Cai L.Y. (2022). Preparation, characterization of naringenin, β-cyclodextrin and carbon quantum dot antioxidant nanocomposites. Food Chem..

[39] Dai K.K., Wu J.Y., Liu X.Y., Wang S.L., Liu Y.H., Li H.H., Wang H.X. (2024). Inclusion complex of quercetin with sulfobutylether β-cyclodextrin: preparation, characterization, antioxidant and antibacterial activities and the inclusion mechanism. RSC Adv..

[40] Zhang L., Qiu J.H., Dai D.L., Zhou Y.C., Liu X., Yao J.F. (2022). Cr-metal-organic framework coordination with Zn2S4 nanosheets for photocatalytic reduction of Cr(VI). J. Clean. Prod..

[41] Yang G.-G., Zhou D.-J., Pan Z.-Y., Yang J., Zhang D.-Y., Cao Q., Ji L.-N., Mao Z.-W. (2019). Multifunctional low-temperature photothermal nanodrug with in vivo clearance, ROS-scavenging and anti-inflammatory abilities. Biomaterials.

[42] Wu Q.S., Yang H.P., Kang L., Gao Z., Ren F.F. (2020). Fe-based metal-organic frameworks as Fenton-like catalysts for highly efficient degradation of tetracycline hydrochloride over a wide pH range: acceleration of Fe(II)/Fe(III) cycle under visible light irradiation. Appl. Catal. B Environ..

[43] Zhou J., Li J., Kan L., Zhang L., Huang Q., Yan Y., Chen Y.F., Liu J., Li S.L., Lan Y.Q. (2022). Linking oxidative and reductive clusters to prepare crystalline porous catalysts for photocatalytic CO2 reduction with H2O. Nat. Commun..

[44] Lu B.Y., Wu X.R., Xiao X., Chen B., Zeng W.H., Liu Y.Q., Lao Z.J., Zeng X.X., Zhou G.M., Yang J.L. (2024). Energy band engineering guided design of bidirectional catalyst for reversible Li-CO2 batteries. Adv. Mater..

[45] Liang H., Liu R.P., Hu C.Z., An X.Q., Zhang X.W., Liu H.J., Qu J.H. (2021). Synergistic effect of dual sites on bimetal-organic frameworks for highly efficient peroxide activation. J. Hazard Mater..

[46] Yu K.H., Wei T.X., Li Z.J., Li J.Y., Wang Z.Y., Dai Z.H. (2020). Construction of molecular sensing and logic systems based on site-occupying effect-modulated MOF-DNA interaction. J. Am. Chem. Soc..

[47] Bao C.S., Wang H., Wang C.Y., Zhang X.H., Zhao X.L., Dong C.L., Huang Y.C., Chen S., Guo P., She X.L., Sun Y.Y., Yang D.J. (2023). Cooperation of oxygen vacancy and FeIII/FeII sites in H2-reduced Fe-MIL-101 for enhanced Fenton-like degradation of organic pollutants. J. Hazard Mater..

[48] Zhang X.N., Ma X.J., Ye Y.Q., Guo C.X., Xu X.J., Zhou J.W., Wang B. (2023). Enhanced photocatalytic hydrogen evolution with a mixed-valence iron metal-organic framework. Chem. Eng. J..

[49] Mao D.J., Yang S.X., Hu Y., He H., Yang S.G., Zheng S.R., Sun C., Jiang Z.F., Qu X.L., Wong P.K. (2023). Efficient CO2 photoreduction triggered by oxygen vacancies in ultrafine Bi5O7Br nanowires. Appl. Catal. B Environ. Energy.

[50] Hao L., Huang H.W., Zhang Y.H., Ma T.Y. (2021). Oxygen vacant semiconductor photocatalysts. Adv. Funct. Mater..

[51] Liu Y.N., Zhao D.J., Yang F., Ye C.H., Chen Z.Y., Chen Y.H., Yu X.M., Xie J.Y., Dou Y., Chang J. (2024). In situ self-assembled Phytopolyphenol-Coordinated intelligent nanotherapeutics for multipronged management of ferroptosis-driven Alzheimer's disease. ACS Nano.

[52] Li L., Zhang M.X., Liu T.T., Li J., Sun S.L., Chen J.J., Liu Z.M., Zhang Z.R., Zhang L. (2022). Quercetin-ferrum nanoparticles enhance photothermal therapy by modulating the tumor immunosuppressive microenvironment. Acta Biomater..

[53] Pan T.N., Yang K., Li J.W., Pang E., Zhao S.J., Xing X.J., Tan Q.X., Wang Q., Yi J.N., Lan M.H. (2023). Self-assembled quercetin-Fe3+ nanoparticles for synergetic near-infrared light-triggered low-temperature photothermal/glutathione-activated chemodynamic therapy. Sci. China Mater..

[54] Megala M., Mayandi J., Rajkumar B.J.M. (2020). Optical absorption study of anthoxanthin based natural dyes for dye sensitized solar cells: experimental and theoretical investigations. Mater. Lett..

[55] Zhu Q.H., Hailili R., Xin Y., Zhou Y.T., Huang Y., Pang X.Z., Zhang K., Robertson P.K.J., Bahnemann D.W., Wang C.Y. (2022). Efficient full spectrum responsive photocatalytic NO conversion at Bi2Ti2O7: Co-effect of plasmonic Bi and oxygen vacancies. Appl. Catal. B Environ..

[56] Wei Z., Wang W.C., Li W.L., Bai X.Q., Zhao J.F., Tse E.C.M., Phillips D.L., Zhu Y.F. (2021). Steering electron-hole migration pathways using oxygen vacancies in tungsten oxides to enhance their photocatalytic oxygen evolution performance. Angew. Chem. Int. Ed..

[57] Yuan R.Y.K., Li Y.Q., Han S., Chen X.X., Chen J.Q., He J., Gao H.W., Yang Y., Yang S.L., Yang Y. (2022). Fe-Curcumin nanozyme-mediated reactive oxygen species scavenging and anti-inflammation for acute lung injury. ACS Cent. Sci..

[58] Gebremariam S.K., Varghese A.M., Reddy K.S.K., AlWahedi Y.F., Dumoée L.F., Karanikolos G.N. (2023). Polymer-aided microstructuring of moisture-stable GO-hybridized MOFs for carbon dioxide capture. Chem. Eng. J..

[59] Monro S., Colón K.L., Yin H.M., Roque J., Konda P., Gujar S., Thummel R.P., Lilge L., Cameron C.G., McFarland S.A. (2019). Transition metal complexes and photodynamic therapy from a tumor-centered approach: challenges, opportunities, and highlights from the development of TLD1433. Chem. Rev..

[60] Mehrgardi M.A., Mofidfar M., Zare R.N. (2022). Sprayed water microdroplets are able to generate HydrogenPeroxide spontaneously. J. Am. Chem. Soc..

[61] Zhou J.R., Wang K., Ding S.S., Zeng L.J., Miao J.Y., Cao Y.H., Zhang X., Tian G., Bian X.W. (2022). Anti-VEGFR2-labeled enzyme-immobilized metal-organic frameworks for tumor vasculature targeted catalytic therapy. Acta Biomater..

[62] Murphy M.P., Bayir H., Belousov V., Chang C.J., Davies K.J.A., Davies M.J., Dick T.P., Finkel T., Forman H.J., Janssen-Heininger Y., Gems D., Kagan V.E., Kalyanaraman B., Larsson N.G., Milne G.L., Nyström T., Poulsen H.E., Radi R., Van Remmen H., Schumacker P.T., Thornalley P.J., Toyokuni S., Winterbourn C.C., Yin H.Y., Halliwell B. (2022). Guidelines for measuring reactive oxygen species and oxidative damage in cells and in vivo. Nat. Metab..

[63] Shi Y.F., Zhang G., Xiang C., Liu C.Z., Hu J., Wang J.H., Ge R.L., Ma H.X., Niu Y.S., Xu Y.H. (2024). Defect-engineering-mediated long-lived charge-transfer excited-state in Fe-Gallate complex improves iron cycle and enables sustainable fenton-like reaction. Adv. Mater..

[64] Cao H., Yang L., Tian R., Wu H.X., Gu Z.P., Li Y.W. (2022). Versatile polyphenolic platforms in regulating cell biology. Chem. Soc. Rev..

[65] Lei Q., He D.F., Ding L.P., Kong F.H., He P.Y., Huang J.D., Guo J.M., Brinker C.J., Luo G.X., Zhu W., Yu Y.L. (2022). Microneedle patches integrated with biomineralized melanin nanoparticles for simultaneous skin tumor photothermal therapy and wound healing. Adv. Funct. Mater..

[66] Kantapan J., Anukul N., Leetrakool N., Rolin G., Vergote J., Dechsupa N. (2021). Iron-quercetin complex preconditioning of human peripheral blood mononuclear cells accelerates angiogenic and fibroblast migration: implications for wound healing. Int. J. Mol. Sci..

[67] Yang S., Zhu Y., Ji C., Zhu H., Lao A., Zhao R., Hu Y., Zhou Y., Zhou J., Lin K., Xu Y. (2024). A five-in-one novel MOF-modified injectable hydrogel with thermo-sensitive and adhesive properties for promoting alveolar bone repair in periodontitis: antibacterial, hemostasis, immune reprogramming, pro-osteo-/angiogenesis and recruitment. Bioact. Mater..

[68] Chen J., Liu Y.J., Cheng G.P., Guo J.H., Du S., Qiu J.M., Wang C., Li C.C., Yang X.F., Chen T.K., Chen Z.B. (2022). Tailored hydrogel delivering niobium carbide boosts ROS-scavenging and antimicrobial activities for diabetic wound healing. Small.

[69] Mandic L., Matkovic M., Baranovic G., Segota S. (2023). The increased release kinetics of quercetin from superparamagnetic nanocarriers in dialysis. Antioxidants.

