# Supplementary material for: Fe-MOF@Quercetin complex with light-controlled redox modulation for tumor wound repair
Source: Mater Today Bio. 2026 Jul 21;39:103484. doi: 10.1016/j.mtbio.2026.103484 (PMC13449421; doi:10.1016/j.mtbio.2026.103484)
Supplement: Supplementary file 1 [file mmc1.docx]

**Supporting Information**

**Figure S1.** Comparative hydrodynamic size distributions and colloidal dispersion characteristics of MOF and MQ particles.

**Figure S2.** Surface electrokinetic potential and charge polarity inversion of MOF and MQ particles.

**Figure S3.** Absorbance of Fe and Qu mixed in different ratio.

**Figure S4.** Fe ions release from MOF and MQ in simulated tumor weak acid environment (RMPI 1640, pH=6.0) and simulated wound neutral environment (HDMEM, pH=7.4), respectively (n=3).

**Figure S5.** The absorbance change of Qu-Fe chelate released by MQ in a simulated tumor weak acid environment (sodium acetate, pH=6.0) and a simulated wound neutral environment (PBS, pH=7.4) within 120 hours (430nm is the default characteristic absorption peak).

**Figure S6.** XPS full spectrum of MOF and MQ.

**Figure S7.** (a) C 1s peak and (b) O 1s peak of MOF and MQ.

**Figure S8.** Qu standard curve (dispersed in ethanol solution, n=4).

**Figure S9.** Comparison of reduction ability among Qu, MOF and MQ. (a) Changes in absorbance of ABTS solution and (b) DPPH solution within 24 hours.

**Figure S10.** ROS generation and scavenging efficiency of the MQ complex.

**Figure S11.** Evaluation of singlet oxygen (^1^O_2_) generation capacity by SOSG.

**Figure S12.** Evaluation of singlet oxygen (^1^O_2_) generation capacity by ESR.

**Figure S13.** Quantitative evaluation of light-triggered generation of reactive oxygen species (ROS) by measuring the absorbance of TMB oxidation products at 652 nm in different treatment groups (Blank, MQ, and MQ+Laser).

**Figure S14.** Representative CLSM images of DCFH-DA stained HUVECs (pH 7.4) cultured with MQ under different treatment conditions such as continuous dark (Laser Off), near-infrared irradiation (Laser On, 10 min), and post-irradiation dark (Laser Off, 10 min) and corresponding quantitative analysis of the mean fluorescence intensity. ***p < 0.001 compared to the Laser On group; ###p < 0.001 compared to the Laser On (10 min) groups.

**Figure S15.** Representative CLSM images and fluorescence intensity analysis showing intracellular ROS levels in B16F10 cells with or without laser irradiation and at different pH environment. Three conditions were investigated such Laser On (10min) at pH 6.0, Laser Off (10min) at pH 6.0 and Laser Off at pH 7.4. *< 0.05, ***p < 0.001 compared to the initial Laser Off; ##p < 0.01, ###p < 0.001 compared to the Laser Off (10 min) groups.

**Figure S16.** Relative mRNA expression levels of pro-apoptotic (Bax, Caspase-3, p53) and pro-inflammatory (TNF-α, IL-6, and IL-1β) genes in B16F10 cells following MQ treatment with or without laser irradiation (660 nm, 0.5 W/cm^2^, 10 min).

**Figure S17.** Relative mRNA expression levels of inflammatory (TNF-α, IL-1β) and collagen synthesis-related (Col1a1, Col3a1) genes in HDF cells under H2O2-induced oxidative stress following MQ treatment in the dark.

**Figure S18.** Effects of MOF and MQ on the proliferation activity of HUVECs cells under oxidative stress for 1, 3, and 5 days (H_2_O_2_=100 μM, n=4).

**Figure S19.** Evaluation of cell migration performance. (a) Effects of MOF and MQ on HUVECs cell migration under oxidative stress. (b) Statistics of Cell migration rate (H_2_O_2_=100 μM, n=4).

**Figure S20.** Evaluation of angiogenesis performance. Effects of MOF and MQ on HUVECs angiogenesis under oxidative stress. (b) Statistics of the number of vascular ring intersections (H_2_O_2_=100 μM, n=4).

**Figure S21.** Effect of the laser irradiation on B16F10 and HUVECs cells. Time-dependent cell proliferation dynamics B16F10 melanoma cells and HUVECs cultured with or without near-infrared laser irradiation (660 nm, 0.5 W/cm^2^, 10 min) over a 5-day incubation period.

**Figure S22.** Methodological control and evaluation of laser-induced intracellular oxidative stress. ESR spectra of B16F10 melanoma cells subjected to singular near-infrared laser irradiation (660 nm, 0.5 W/cm^2^, 10 min) utilizing BMPO (5-Tert-butoxycarbonyl-5-methyl-1-pyrroline-N-oxide) to monitor intracellular ROS dynamics.

**Figure S23.** Histological validation of effective transdermal penetration by the composite microneedle array.

**Figure S24.** In vitro biodegradation kinetics of the MQ@MNs. The progressive mass loss profile of the composite microneedle patches immersed in PBS (pH 7.4) at 37 °C over a 96-hour duration.

**Figure S25.** Vascular node counts and fluorescence intensity statistics of CD31.

**Figure S26.** Dynamic monitoring of body weight in the Blank and MQ@MNs groups over the 12-day treatment period in normal mouse.

**Figure S27.** Quantitative analysis of core hepatic function markers (ALT and AST) in mouse serum at the treatment endpoint.

**Figure S28.** Quantitative analysis of core renal function markers (BUN and CREA) in mouse serum at the treatment endpoint.

**Figure S29.** H&E staining images of the main organ tissues (heart, liver, spleen, lung, and kidney) of mice after 12 days of treatment.

**Figure S30.** In vivo photothermal evaluation and synergistic tumor ablation efficacy. (a) Representative time-dependent macroscopic photographs of the melanoma-bearing mice in the Blank, MQ@MNs+PTT groups and MQ@MNs+PDT. (b) Optical images of the completely excised tumors at the endpoint of the observation period. (c) Real-time infrared thermal mapping of the tumor sites under corresponding continuous laser excitation. (d) Corresponding temporal temperature evolution trajectories at the targeted tumor regions over the 10-minute irradiation duration.

**Table S1.** Qu drug loading rate calculation in MQ (n=4).

**Table S2.** Oligonucleotide primer sequences utilized for quantitative real-time PCR (RT-qPCR) amplification.

| Gene | Species | Primer Orientation | Sequence (5’ → 3’) |
| --- | --- | --- | --- |
| Gsta1 | Human | Forward | TGT TGA TTG TTT GCC TGA AAT T |
|  |  | Reverse | GTT AAA CGC TGT CAC CCG TCC T |
| Bax | Mouse | Forward | TCG TCC ATC GAG GAT GAC TTC |
|  |  | Reverse | AAC ACC ACA ATT AAG GCA GGG |
| Caspase-3 | Mouse | Forward | CTC GCT CTG GTA CGG ATG TG |
|  |  | Reverse | TCC CAT AAA TGA CCC CTT CAT CA |
| p53 | Mouse | Forward | CCC CGG TTG CTT CTG GTA G |
|  |  | Reverse | TCA CCT GTG TGT AAG TCA CTC T |
| TNF-α | Mouse | Forward | GCT GGG CAA CAA GTA CGT G |
|  |  | Reverse | GTG CTT TCC ACA TCG GTC TAT G |
| IL-6 | Mouse | Forward | CTG CAA GAG ACT TCC ATC CAG |
|  |  | Reverse | AGT GGT ATA GAC AGG TCT GTT GG |
| IL-1β | Mouse | Forward | ATG ATG GCT TAT TAC AGT GGC AA |
|  |  | Reverse | GTC GGA GAT TCG TAG CTG GA |
| TNF-α | Human | Forward | GGG TTG GGC AAC AAG TAT GTC |
|  |  | Reverse | GGT GTC ATC TCG GAG GTA ATT CA |
| IL-1β | Human | Forward | ATG ATG GCT TAT TAC AGT GGC AA |
|  |  | Reverse | GTC GGA GAT TCG TAG CTG GA |
| Col1a1 | Human | Forward | GAG GGC CAA GAC GAA GAC ATC |
|  |  | Reverse | CAG ATC ACG TCA TCG CAC AAC |
| Col3a1 | Human | Forward | GGA GCT GGC TAC TTC TCG C |
|  |  | Reverse | GGG AAC ATC CTC CTT CAA CAG |
| GAPDH | Mouse | Forward | AAT GGA TTT GGA CGC ATT GGT |
|  |  | Reverse | TTT GCA CTG GTA CGT GTT GAT |
| GAPDH | Human | Forward | TGG CAA ATT CCA TGC AC |
|  |  | Reverse | CCA TGG TGG TGA AGA CGC |
